# Six years progression of exercise capacity in subjects with mild to moderate airflow obstruction, smoking and never smoking controls

**DOI:** 10.1371/journal.pone.0208841

**Published:** 2018-12-26

**Authors:** Fernanda Machado Rodrigues, Matthias Loeckx, Miek Hornikx, Hans Van Remoortel, Zafeiris Louvaris, Heleen Demeyer, Wim Janssens, Thierry Troosters

**Affiliations:** 1 Department of Rehabilitation Sciences, KU Leuven – University of Leuven, Leuven, Belgium; 2 Department of Respiratory Diseases, University Hospitals Leuven, Leuven, Belgium; 3 Department of Physiotherapy, LUNEX University, Differdange, Luxembourg; 4 Department of Cardiovascular Sciences, University Hospitals Leuven, Leuven, Belgium; 5 Centre for Evidence-Based Practice, Belgian Red Cross-Flanders, Mechelen, Belgium; 6 Department of Chronic Diseases, Metabolism and Ageing (CHROMETA), University Hospital Leuven, KU Leuven, Leuven, Belgium; National Yang-Ming University, TAIWAN

## Abstract

**Background:**

Exercise capacity is an important feature in patients with COPD. Its impairment drives disability and dependency for daily activities performance. This study evaluated the six years change in exercise capacity in subjects with airflow obstruction and compared this to subjects without airflow obstruction, with and without a smoking history.

**Methods:**

Cardiopulmonary exercise tests (CPET) were repeatedly performed during a six years follow up period. Peak oxygen uptake (VO_2_peak), work rate (WRpeak), heart rate (HRpeak), minute ventilation (VEpeak), respiratory exchange ratio (RERpeak) and ventilatory reserve (VE/MVV) were collected as effort dependent outcomes. The slopes of oxygen uptake, ventilatory and mechanical efficiency (OUES, ΔVE/ΔVCO_2_ and ΔVO_2_/ΔWR) were collected as effort independent outcomes.

**Results:**

One hundred and thirty-eight subjects were included. Thirty-eight presented airflow obstruction (63±6 years, 74% men, FEV_1_ 90±15%_pred_), 44 had a smoking history but no airflow obstruction (61±5 years, 61% men, FEV_1_ 105±15%_pred_) and 56 had never smoked (61±7 years, 57% men, FEV_1_ 117±18%_pred_). At baseline, the airflow obstruction group had slightly worse exercise capacity in comparison to the never smoking control group, in absolute terms and expressed as percentage of the predicted value (VO_2_peak = 27±5 versus 32±8 ml/min/kg, p<0.01; 112±29 versus 130±33%_pred_, p = 0.04). Most exercise variables showed a statistically significant yearly deterioration, with exception of VE/MVV, ΔVE/ΔVCO_2_ and ΔVO_2_/ΔWR. The yearly decline in VO_2_peak and OUES was not faster in subjects with airflow obstruction than in smoking and never smoking controls (VO_2_peak -67 (9) versus -76 (9) ml/min, p = 0.44 and versus -58 (9), p = 0.47; OUES -32 (11) versus -68 (10), p = 0.03 and versus -68 (13), p = 0.03).

**Conclusions:**

With exception of VO_2_peak, effort dependent variables deteriorated faster in subjects with airflow obstruction compared to never smoking controls. The deterioration of effort independent variables, however, was not accelerated in the airflow obstruction group compared to controls.

## Introduction

Chronic obstructive pulmonary disease (COPD) is characterized by airflow obstruction and is one of the leading causes of morbidity and mortality worldwide [1]. The major risk factor for developing COPD is cigarette smoking, although not all smokers develop the disease [2]. Exercise capacity is known to be reduced both in smokers [3] and in patients with COPD [4]. Exercise tolerance is crucial in the management of patients with symptomatic COPD since its impairment drives disability, decreased social and recreational participation and even dependency on others for performing daily activities in the advanced stages [5].

The cardiopulmonary exercise test (CPET) is the gold standard test to assess exercise capacity. It is useful in providing information on prognosis, exercise prescription risk and, to a lesser extent, the assessment of treatment effects [6]. When incremental exercise tests are used, peak exercise responses, including peak oxygen uptake (VO_2_peak), peak work rate (WRpeak), peak heart rate (HRpeak) and peak ventilation (VEpeak), are typically reported. The VO_2_peak is widely accepted to be a reliable measure of cardiorespiratory fitness, in healthy subjects and in patients with COPD [6].

Beyond the outcomes at peak exercise, other outcomes can be extracted from this test. These outcomes provide a less effort dependent assessment [7, 8] and more information about the responses to exercise. Breath by breath analysis allows calculation of the so called ‘efficiency slopes’. 1) The oxygen uptake efficiency slope (OUES) reflects the rate of increase of the logarithmically transformed ventilation in response to a given increase in oxygen uptake and it correlates highly with VO_2_peak in cross sectional studies [9]. The OUES is a prognostic marker in patients with chronic heart failure and coronary artery disease [10, 11], which both are common comorbidities of COPD [12]. 2) The ventilatory efficiency slope (ΔVE/ΔVCO_2_), which represents the increase in ventilation for a given rise in carbon dioxide production, is increased when ventilation perfusion mismatching occurs, in case of diffusion abnormalities or with an excess dead space ventilation. The excess in ventilation may lead to more rapid dynamic hyperinflation and reduced exercise capacity in COPD [13, 14]. 3) Lastly, the mechanical efficiency (ΔVO_2_/ΔWR) is also of interest as it provides information on the capacity of the musculoskeletal system to generate power from the aerobic energy pathways [15]. While these slopes are of interest in clinical practice, little is known about their changes over time.

Only a few studies have used VO_2_peak to investigate the deterioration of exercise capacity in patients with moderate to severe COPD [16, 17]. Little is known on how the less effort dependent variables change over time [18]. This is particularly important in smokers with some extend of airflow obstruction, where the impact of cigarette smoking, and potential development of clinical COPD, may have greater influence on exercise capacity. Investigating the longitudinal progression of exercise capacity parameters in COPD, especially in its early stages, might provide important insight on the course of the disease and a better understanding of disease progression, if any. With this aim, we have performed a six years case-control cohort study of subjects with a significant smoking history with mild to moderate airflow obstruction and compared them with smoking and never smoking controls without airflow obstruction.

The aim of this study was to describe and compare the progression of effort dependent and effort independent outcomes obtained from a maximal incremental exercise test in subjects with mild to moderate airflow obstruction, smoking and never smoking controls. We hypothesized that the airflow obstruction group would present faster deterioration in exercise related outcomes than the never smoking and smoking control groups. This, in turn, would potentially lead to the impaired exercise capacity present in patients with more advanced COPD previously reported in literature.

## Methods

### Participants

Three groups were included: 1) subjects with mild to moderate airflow obstruction (airflow obstruction, reference group), 2) ex-/smokers with a significant smoking history but without airflow obstruction (smoking controls), and 3) never or ex-smokers with a marginal smoking history (never smoking controls). As previously described [19], subjects with airflow obstruction and smoking controls were mainly recruited from a population based cohort of (ex-) smokers. The never smoking control group was composed of co-workers of the University Hospital and of participants of an organization for elderly in the city of Leuven. For inclusion in the trial, subjects were between 40 and 80 years old. Subjects from the smoking control and airflow obstruction groups had a smoking history of at least 10 pack years. Both control groups included subjects with normal spirometry. Subjects with mild to moderate airflow obstruction had a post bronchodilator FEV_1_/FVC (forced expiratory volume in one second and forced vital capacity ratio) lower than 70% [1] and an FEV_1_ (forced expiratory volume in one second) higher than 60% of the predicted normal value. Importantly, none of these subjects had a history of medical care by a chest physician and the vast majority of patients was never formally diagnosed with COPD at the start of the study. Exclusion criteria were respiratory diseases other than COPD, use of corticosteroids in the 6 weeks preceding the inclusion and important orthopedic or neurologic problems that could interfere with the physical tests or normal performance of physical activity.

### Design

This study is part of the Rainbow trial, a prospective, case-control, observational trial that had a six years follow up period and aimed to investigate the prevalence, severity and incidence of systemic consequences in newly detected patients with mild and moderate COPD. The Rainbow trial was approved by the ethics committee of the University Hospital Leuven (Research Ethics Committee UZ/KU Leuven—B3220096387) and was registered on ClinicalTrials.gov (NCT01314807). Subjects were included between June 2009 and February 2012 after providing written informed consent. The last patient performed his last visit in December 2017.

The main variables for the present analyses were peak oxygen uptake (VO_2_peak), peak work rate (WRpeak), peak heart rate (HRpeak), peak minute ventilation (VEpeak), ventilatory reserve (VE/MVV), peak respiratory exchange (RERpeak) and the slopes of oxygen uptake, ventilatory and mechanical efficiency (OUES, ΔVE/ΔVCO_2_ and ΔVO_2_/ΔWR, respectively). These were investigated at baseline and after one, two, three and six years in the airflow obstruction and smoking control groups and at baseline and after three and six years in the never smoking control group.

### Measurements

#### Anamnesis and characterization

At inclusion and at every follow up visit, a comprehensive interview was performed to assess the clinical and smoking status. Lung function and diffusion capacity were assessed according to the European Respiratory Society recommendations [20, 21]. Subjects with airflow obstruction were further classified according to GOLD spirometric criteria into stage 1 (FEV_1_> 80% pred) or 2 (80> FEV_1_> 50% pred) [1]. Functional capacity was evaluated with the six minutes walking test [22] and quadriceps force as maximal voluntary contraction measured with the computerized dynamometer Biodex system 4 pro [23, 24]. Physical activity was included in this analysis for characterization. Participants wore the SenseWear Pro 2 Armband (Bodymedia, Pittsburgh, PA) for seven days following each visit. A measure was considered valid if the device was worn for at least four days, during at least eight hours per day (between 07:00 AM and 20:00 PM) [25]. The valid days were averaged to obtain the mean amount of steps per day and total time spent in moderate to intense activities (MVPA defined as an intensity above 3 METS) [26].

#### Maximal incremental exercise test

Prior to each CPET, subjects performed spirometry to determine Forced Expiratory Volume in one second (FEV_1_), and Forced Vital Capacity (FVC). Maximal Voluntary Ventilation (MVV) was assessed as the maximal volume that could be generated from deep and fast breathing for 12 seconds [20]. Over the whole duration of the study, the maximal incremental test was performed on the same exercise set-up consisting of a cycle ergometer (Ergometrics 900, Ergoline, Bitz, Germany). The test protocol consisted of minimally two minutes of rest followed by a stage of unloaded cycling for three minutes. Then, an initial load of 20 watts was imposed followed by consecutive increases of 20 watts every minute until volitional exhaustion. The CPET protocol was standardized for all individuals and across all visits. Oxygen uptake (VO_2_), carbon dioxide output (VCO_2_) and minute ventilation (VE) were measured breath by breath and data were exported using a 10 seconds average (Vmax series, SensorMedics, Anaheim, CA). All tests were supervised by a medical doctor. Blood pressure was assessed every 2 minutes, peripheral arterial oxygen saturation and a 12 led electrocardiogram was continuously measured. At the end of the test, the level of reported dyspnea and leg fatigue was assessed using the modified 10 points Borg scale. After the test, data were exported with ten seconds averages to an excel file for further semi-automated processing.

In the present study, peak oxygen uptake (VO_2_peak) was defined as the highest 20 seconds average of VO_2_ achieved during the test. Peak work rate (WRpeak), peak minute ventilation (VEpeak), peak heart rate (HRpeak) and peak respiratory exchange ratio (RERpeak) were calculated as the 20 seconds average of data points using the same time points as VO_2_peak. Ventilatory reserve was expressed as the ratio of VEpeak over MVV (VE/MVV). The higher the VE/MVV, the lower the ventilatory reserve. In case of missing data for MVV (2.3% of total data), MVV was estimated from FEV_1_, using the average of the ratio of MVV and FEV_1_ in each of the other visits each individual subject (the average ratio ranged from 32 to 48 in these subjects). The efficiency slopes were calculated using data raw data from the test after excluding the undesired inflections in VE and VO_2_ that normally occur after the initial loaded stage and after the ventilatory compensation point. To this end, data up to the first loaded stage (unloaded pedaling and 20 watts) were automatically deleted, as well as data obtained during the last 25% of the test, if RERpeak was equal or higher than 1.1. If RERpeak was lower than 1.1, no data points at the end of the test were deleted. An alternative approach with deleting the last 10% of the exercise tests yielded similar results. A linear regression analysis was performed between VO_2_ and the 10 logarithmic transformation of VE to calculate OUES [9]. ΔVE/ΔVCO_2_ was calculated from the linear regression between VCO_2_ and VE [27] and ΔVO_2_/ΔWR was calculated from the linear regression between VO_2_ and work rate (WR) [28] in every patient, for every test separately. Tests were considered valid and only included in the study when the p values of the F statistic originated from all efficiency slopes were lower than 0.05 [17]. Whereas all exercise related variables were physiologically related and they do not represent the work from a single body compartment, the results were presented in categories to provide a comprehensive overview. ‘Comprehensive measures of cardiovascular fitness’ comprised VO_2_peak, HRpeak and OUES, ‘Outcomes related to pulmonary ventilation’ were VEpeak, VE/MVV and VE/ΔVCO_2_, ‘measures related to muscle work’ included WRpeak and ΔVO_2_/ΔWR and ‘effort indicators’ were RERpeak and BORG scores for dyspnea and fatigue.

#### Exploratory measures

Information regarding smoking exposure and use of medication were collected during each visit and used in exploratory analyses. Cardiovascular and metabolic risk factors and comorbidities were also measured for exploratory analyses. Obesity, dyslipidemia, hypertension, arterial stiffness, atherosclerosis, pre diabetes, cardiac dysfunction, peripheral arterial disease, diabetes and osteoporosis were objectively measured as described elsewhere [19].

### Statistical analysis

Data handling and statistical analyses were performed with SAS 9.4 (SAS Institute Inc, Cary, North Carolina, USA). Subjects were included in the analyses if they had CPET results of, at least, baseline and at the six years follow up visit. For visual representation of the deterioration in the outcomes of interest, measurements from each visit were averaged by group (PROC means) and plotted in graphs using GraphPad Prism version 4.01 (GraphPad Software Inc., San Diego, California, USA).

Comparison of continuous data from baseline characteristics were performed by ANOVA or the non-parametric equivalent (Kruskal-Wallis). Frequency distribution of gender and smoking status, as categorical data, was compared with a chi-square test. Post hoc tests were performed including a correction for multiple comparisons.

As the main analysis, a mixed model was built (PROC mixed) for evaluating possible differences in the yearly rate of decline in the outcomes of interest among groups. This was investigated with the interaction factor between group and time. Group and subject identification were added as class variables, time as continuous variable, and intercept (initial potency) and slopes (degradation rate), as random effects. The airflow obstruction group was indicated as reference group in the model. The analysis regarding HRpeak was performed including all subjects and excluding those who were under beta blocker therapy in any of the CPETs.

As a sensitivity analysis and in order to be included in the exploratory analysis, for every subject, a simple linear regression analysis (PROC autoreg) based on all available data points provided the estimate of baseline (‘intercept’) and the individual yearly change (‘slope’). The yearly rate of change (slope) was calculated and expressed as absolute value as well as a percentage of the estimated baseline. A paired t test was applied to verify if the rate of change was different from zero and, therefore, statistically significant. The comparison of the baseline and the individual yearly change among the three groups was performed with a one-way analysis of variance (ANOVA) test.

The following exploratory analyses were performed: 1) the main mixed model analysis was repeated including the covariates age, gender, height and weight in order to verify possible interferences in the main results. 2) The rate of decline in exercise related variables was compared between subjects in the airflow obstruction group who were under maintenance respiratory pharmacotherapy (long acting beta agonists, long acting anticholinergics and inhaled corticosteroids) during follow-up and those who were not. 3) The rate of deterioration was compared between subjects who were actively smoking or not during the study, from those who had an important smoking history. 4) The potential impact of cardiovascular and metabolic risk factors and comorbidities on the deterioration of exercise related outcomes was investigated comparing the yearly rate of decline in the exercise related between subjects ever or never presenting the risk factors or comorbidities. The comparisons between groups created in the exploratory analyses were performed with an independent T test on the rate of decline obtained from the linear simple linear regression analysis (slopes) or with a Chi-square or Fisher exact test for the frequency distribution of comorbidities and risk factors.

## Results

### Characteristics

**Fig 1:**
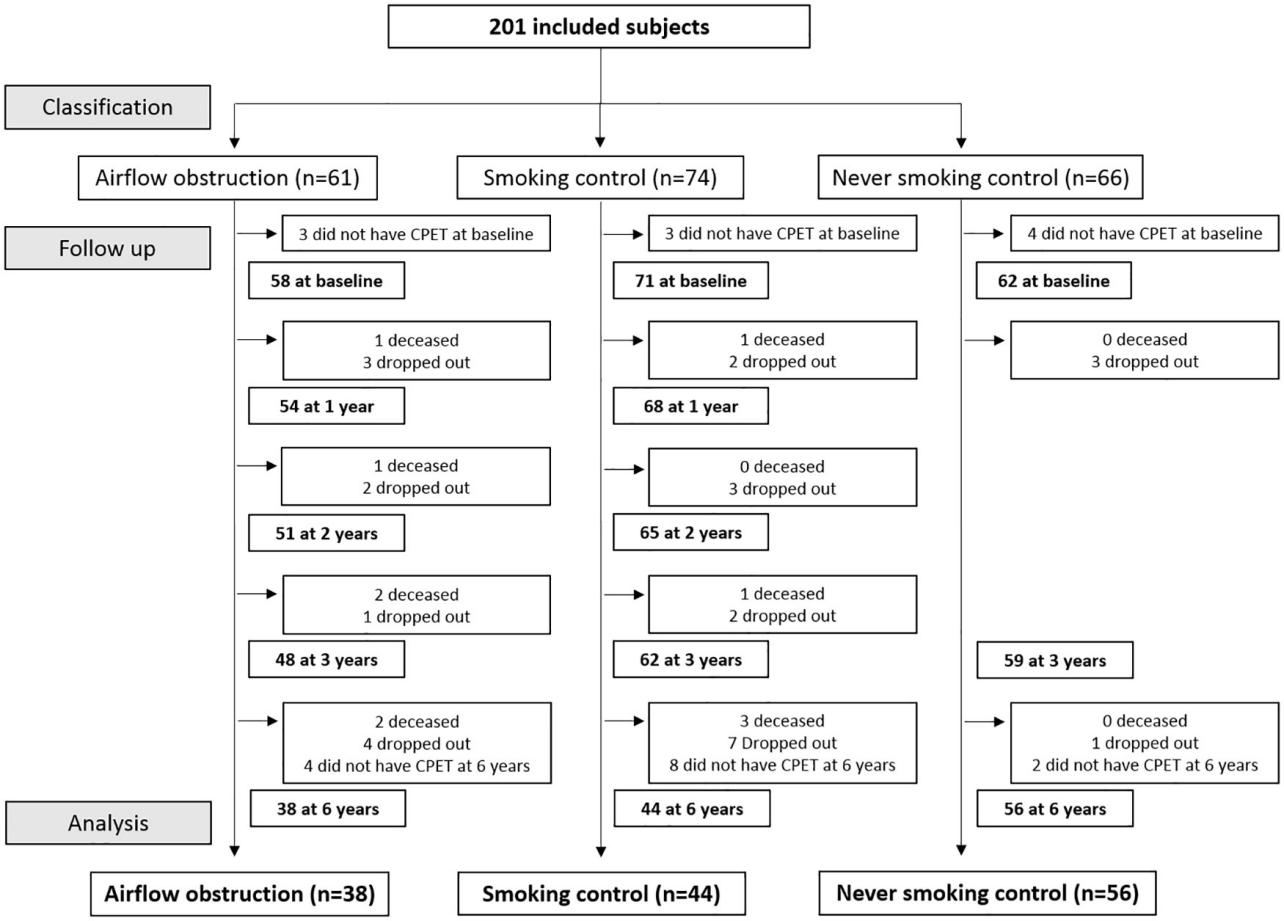
Flow chart of inclusion and follow up.

Two hundred and one subjects were included in the Rainbow study. Ten subjects did not perform CPET at baseline, 11 died, 28 dropped out from the study and 14 did not want or had medical contraindications to perform a CPET at the six years follow up visit. One hundred and thirty-eight subjects had CPET results, at least, at baseline and at six years follow up. A total of 526 CPETs were included in this study (average of 3.8 tests per subject). Fig 1 shows the flow chart of inclusion and follow up. The characteristics of subjects initially included in the Rainbow trial and those who were maintained in this study can be found in the online supplement (S1 Table). The baseline characteristics of the subjects included in the present study can be found in Table 1. Overall, the three groups were well matched for age, body mass index (BMI) and gender distribution. Four subjects in the never smoking control group had an irrelevant smoking history (range between five and seven pack years) and did quit their occasional smoking five to twenty-five years prior to inclusion. The smoking history of subjects in the airflow obstruction and smoking control groups was not statistically different and, in both groups, around half of the sample was not smoking during the study. Seventy nine percent of subjects with airflow obstruction were classified in stage 1 and 21% in stage 2 according to the spirometric classification of severity proposed by GOLD [1]. At baseline, subjects with airflow obstruction showed a statistically significant lower functional exercise capacity than never smoking controls (p<0001).

**Table 1:** Baseline characteristics.

	Airflow obstruction (n = 38)	Smoking control (n = 44)	Never smoking control (n = 56)	ANOVA p
Age (years)	63±6	61±5	61±7	0.35
Gender [n (% men)]	28 (74)	27 (61)	32 (57)	0.25
BMI (kg/m^2^)	27±4	26±4	25±3	0.11
Body weight (kilogram)	79±14	77±15	74±14	0.23
Smoking hystory (pack year)	45±22	38±23	0±2#†	<0.0001
Not smoking during study [n (%)]	17 (45)	24 (55)	56 (100) #†	<0.0001
GOLD spirometric classification				
Stage 1 [n (%)]	30 (79)	-	-	-
Stage 2 [n (%)]	8 (21)	-	-	-
6MWD (meter)	606±75	614±56	669±75#†	<0.0001
6MWD (% predicted)	92±10	93±8	101±10#†	<0.0001
Quadriceps force (% predicted)	101±24	97±17	112±26†	<0.01
Steps per day	8699±3974	9640±3083	10285±3367	0.10
Time in MVPA (min)	98±71	115±64	114±54	0.41

Data are expressed as mean±std or number (%). BMI = body mass index, kg/m^2^ = kilogram per square meter, GOLD = Global Initiative for Obstructive Lung Disease, 6MWD = six minutes walking distance, MVPA = moderate to intense activities (above 3 METS), min = minute. Missing values: Airflow obstruction group– 1 for 6MWD; Smoking control group– 2 for 6MWD, 3 for quadriceps force and 8 for physical activity; Never smoking control group –1 for 6MWD, 3 for quadriceps force and 5 for physical activity.

^#^ = statistically different from airflow obstruction group.

^†^ = statistically different from smoking control group.

The baseline characteristics of the subgroups of subjects who were ever or never under beta blocker therapy during the visits are available in S2 Table.

Table 2 provides an overview of the lung function and exercise related variables, at baseline and at six years follow up, in the three groups. As anticipated, at baseline, subjects in the airflow obstruction group had the lowest lung function, but lung function impairment was, on average, mild (FEV_1_ 90±15% predicted). This group also presented statistically lower peak exercise capacity compared to never smoking controls. The never smoking control group attained higher HRpeak, WRpeak and VO_2_peak, but not higher OUES, compared to the group with airflow obstruction. While VEpeak was similar among groups, subjects with airflow obstruction presented lower ventilatory reserve at peak exercise as a consequence of their reduced MVV (VE/MVV = 76±16% versus 66±13% and 62±13%, p<0.0001) and had a worse ΔVE/ΔVCO_2_ (29.0±4.8 versus 26.7±3.6 and 26.1±3.9, p<0.01) than the smoking and healthy controls. The median value of dyspnea and fatigue at peak exercise were relatively low. When considering the single highest symptom at the end of the test (either dyspnea or fatigue) this was 6 [5–7] in the airflow obstruction group, 7 [5–7] in smoking controls and 5 [4–7] in never smoking controls (p<0.01 between smoking and never smoking control). At six years, VO_2_peak and WRpeak were statistically different between smoking and never smoking controls, HRpeak differed among all groups and RERpeak was different between the airflow obstruction and the never smoking control groups.

**Table 2:** Baseline and six years lung function and exercise related measurements.

	Airflow obstruction (n = 38)		Smoking control (n = 44)		Never smoking control (n = 56)		ANOVA p	
	Baseline	6 years	Baseline	6 years	Baseline	6 years	Baseline	6 years
Lung function								
FEV_1_/FVC (%)	64±6†§	61±7†§	76±4	74±5	77±5	77±6	<0.0001	<0.0001
FEV_1_ (liter)	2.64±0.62†§	2.46±0.63†§	3.04±0.73	2.92±0.81	3.33±0.75	3.17±0.74	<0.0001	<0.0001
FEV_1_ (% predicted)	90±15	90±16	105±15	108±20	117±18*	119±18*	<0.0001	<0.0001
TL,CO (ml/min/kPa)	7.13±1.82	6.48±1.81	7.50±1.77	7.03±1.64	8.41±2.149#	7.96±1.99#†	<0.01	<0.001
TL,CO (% predicted)	81±18	78±18	86±13	85±13	97±17#†	96±16#†	<0.0001	<0.0001
FRC (liter)	4.21±0.91†§	4.42±0.97†§	3.51±0.60	3.58±0.63	3.57±0.78	3.58±0.75	<0.0001	<0.0001
FCR (% predicted)	126±24†§	131±2†§	109±18	111±18	112±20	111±19	<0.001	<0.0001
Cardiovascular fitness								
VO_2_peak (ml/min/kg)	27±5	22±5	30±7	23±5	32±8#	27±8#†	<0.01	<0.001
VO_2_peak (% predicted)	112±29	100±26	123±34	110±31	130±33#	121±34#	0.04	<0.01
HRpeak (beats/min)	141±17	121±17	149±20	133±20	153±17#	143±22*	<0.01	<0.0001
HRpeak (beats/min)—no βBlocker	145±13	121±17	154±16	133±20	156±15#	143±22*	0.01	<0.0001
OUES (slope)	2547±613	2347±580	2642±619	2269±500	2763±783	2355±779	0.32	0.78
Pulmonary ventilation								
VEpeak (l/min)	81±22	60±15	81±24	63±21	82±24	69±23	0.95	0.14
VE/MVV (%)	76±16†§	66±15	66±13	58±15	62±13	62±16	<0.0001	0.06
ΔVE/ΔVCO_2_ (slope)	29.0±4.8†§	29.9±4.9†§	26.7±3.6	27.6±3.9	26.1±3.9	27.0±3.5	<0.01	<0.01
Muscle work								
WRpeak (watt)	150±35	112±32	163±42	125±34	182±52#	156±56#†	<0.01	<0.0001
WRpeak (% predicted)	95±20	79±27	109±28	93±30	126±35#†	117±36#†	<0.0001	<0.0001
ΔVO_2_/ΔWR (slope)	10.91±1.40	11.42±1.83	11.45±1.76	10.73±2.24	10.63±1.50†	10.72±1.72	0.04	0.17
Effort indicators								
RERpeak	1.15±0.10	1.07±0.09	1.15±0.09	1.11±0.08	1.16±0.12	1.13±0.09#	0.77	0.02
Dyspnea (BORG score)	5 [4–7]	4 [3–5]	5 [3–7]	4 [3–5]	4 [4–5]	4 [3–5]	0.41	0.64
Fatigue (BORG score)	5 [4–7]	4 [4–7]	5.5 [4–7]	4 [4–5]	5 [3–6]†	4 [3–5]	0.03	0.08

Data are expressed as mean±sd or median [interquartile range]. FEV_1_ = forced expiratory volume in one second, TL,CO = diffusion capacity for carbon monoxide, ml/min/kPa = milliliter per minute per kilopascal, FRC = functional residual capacity, VO_2_peak = peak oxygen uptake, ml/min/kg = milliliter per minute per kilogram, WRpeak = peak work rate, HRpeak = peak heart rate. ‘no βBlocker’ refers to the subgroups of subjects who were not under beta blocker medication at any of the visits (airflow obstruction n = 23, Smoking control n = 32, Never smoking control n = 43). See S2 Table for baseline comparisons of this group. VEpeak = peak minute ventilation, VE/MVV = ventilatory reserve, RERpeak = peak respiratory exchange ratio, OUES = oxygen efficiency slope, ΔVE/ΔVCO_2_ = ventilatory efficiency slope, ΔVO_2_/ΔWR = mechanical efficiency. Missing values: BASELINE: Airflow obstruction group– 2 for BORG scores; Smoking control group– 1 for TL,CO, 2 for BORG scores, Never smoking control group– 2 for TL,CO and BORG scores. 6 YEARS: Airflow obstruction group– 1 for BORG scores and for HRpeak; Smoking control group– 1 for TL,CO, FRC and BORG scores; Never smoking control– 1 for BORG scores.

* = statistically different among all groups;

^#^ = statistically different from airflow obstruction group;

^†^ = statistically different from smoking control group;

^§^ = statistically different from never smoking control group.

### Longitudinal progression of outcomes of interest

Results from the mixed model analysis are presented in Table 3. These results were confirmed with the linear regression analyses presented in S3 Table.

**Table 3:** Estimated yearly rate of the decline of the exercise related outcomes.

	Group	Yearly change	P value Interaction effect
Cardiovascular fitness			
VO_2_peak (ml/min)	Airflow obstruction (ref)	-67 (9)*	-
	Smoking control	-76 (9)*	0.44
	Never smoking control	-58 (9)*	0.47
VO_2_peak (ml/min/kg)	Airflow obstruction (ref)	-0.79 (0.11)*	-
	Smoking control	-1.04 (0.12)*	0.18
	Never smoking control	-0.81 (0.13)*	0.94
HRpeak (beats/min)	Airflow obstruction (ref)	-2.94 (0.46)*	-
	Smoking control	-2.55 (0.35)*	0.50
	Never smoking control	-1.63 (0.36)*	0.02
HRpeak (beats/min)	Airflow obstruction (ref)	-3.17 (0.55)*	-
- no βBlocker	Smoking control	-2.44 (0.40)*	0.24
	Never smoking control	-1.39 (0.34)*	<0.01
OUES (slope)	Airflow obstruction (ref)	-32 (11)*	-
	Smoking control	-68 (10)*	0.03
	Never smoking control	-68 (13)*	0.03
Pulmonary ventilation			
VEpeak (l/min)	Airflow obstruction (ref)	-3.24 (0.47)*	-
	Smoking control	-2.85 (0.35)*	0.47
	Never smoking control	-2.21 (0.33)*	0.05
VE/MVV (%)	Airflow obstruction (ref)	-1.4 (0.5)*	-
	Smoking control	-1.3 (0.3)*	0.84
	Never smoking control	-0.1 (0.3)	0.02
ΔVE/ΔVCO_2_ (slope)	Airflow obstruction (ref)	0.09 (0.07)	-
	Smoking control	0.11 (0.08)	0.89
	Never smoking control	0.16 (0.07)*	0.50
Muscle work			
WRpeak (watt)	Airflow obstruction (ref)	-6.31 (0.77)*	-
	Smoking control	-6.42 (0.59)*	0.91
	Never smoking control	-4.21 (0.50)*	0.02
ΔVO_2_/ΔWR (slope)	Airflow obstruction (ref)	0.091 (0.060)	-
	Smoking control	-0.102 (0.070)	0.02
	Never smoking control	0.013 (0.048)	0.35
Effort indicators			
RERpeak	Airflow obstruction (ref)	-0.011 (0.003)*	-
	Smoking control	-0.006 (0.002)*	0.22
	Never smoking control	-0.006 (0.003)*	0.23
Dyspnea (BORG score)	Airflow obstruction (ref)	-0.071 (0.057)	-
	Smoking control	-0.148 (0.061)*	0.36
	Never smoking control	-0.090 (0.050)	0.80
Fatigue (BORG score)	Airflow obstruction (ref)	-0.110 (0.064)	-
	Smoking control	-0.169 (0.051)*	0.40
	Never smoking control	-0.131 (0.048)*	0.73

Data are expressed as mean (standard error). VO_2_peak = peak oxygen uptake, HRpeak = peak heart rate. ‘no βBlocker’ refers to the subgroups of subjects who were not under beta blocker medication at any of the visits (Airflow obstruction n = 23, Smoking control n = 32, Never smoking control n = 43). OUES = oxygen efficiency slope, VEpeak = peak minute ventilation, VE/MVV = ventilatory reserve, ΔVE/ΔVCO_2_ = ventilatory efficiency slope, WRpeak = peak work rate, ΔVO_2_/ΔWR = mechanical efficiency, RERpeak = peak respiratory exchange ratio.

* = statistically significant deterioration.

#### Comprehensive measures of cardiovascular fitness

VO_2_peak, HRpeak and OUES presented a significant yearly deterioration in all groups. The interaction effect shows a statistically faster deterioration in the airflow obstruction group for HRpeak (p = 0.02 in the entire sample and p <0.01 in the sample not taking beta blocker medication) compared to the never smoking group. For VO_2_peak there was no statistically significant difference in the deterioration rate among groups (p>0.05 for all). However, the airflow obstruction group presented a statistically slower deterioration in OUES compared to both control groups (p = 0.03 for both).

#### Outcomes related to pulmonary ventilation

There was a trend for faster deterioration of VEpeak (p = 0.05) in the airflow obstruction group compared to the never smoking control. The VE/MVV decreased faster in the airflow obstruction group, reaching statistically significance (p = 0.02). The rate of change of VE/ΔVCO_2_ did not differ among groups (p = 0.50) and only the never smoking control group deteriorated statistically significant over time.

#### Measures related to muscle work

WRpeak decreased significantly over time in all groups and this decrease was faster in the airflow obstruction compared to never smoking control groups (p = 0.02). Mechanical efficiency (ΔVO_2_/ΔWR) deteriorated faster in the smoking control compared to the airflow obstruction group (p = 0.02). However, the change over time was not statistically significant in any of the groups.

#### Effort indicators

RERpeak decreased significantly in all groups, without statistical difference among groups. In line, the data showed a slight reduction in peak symptoms of fatigue in both control groups and a slight reduction in dyspnea in smoking controls. The changes in symptoms changes over time were not different among groups.

**Fig 2:**
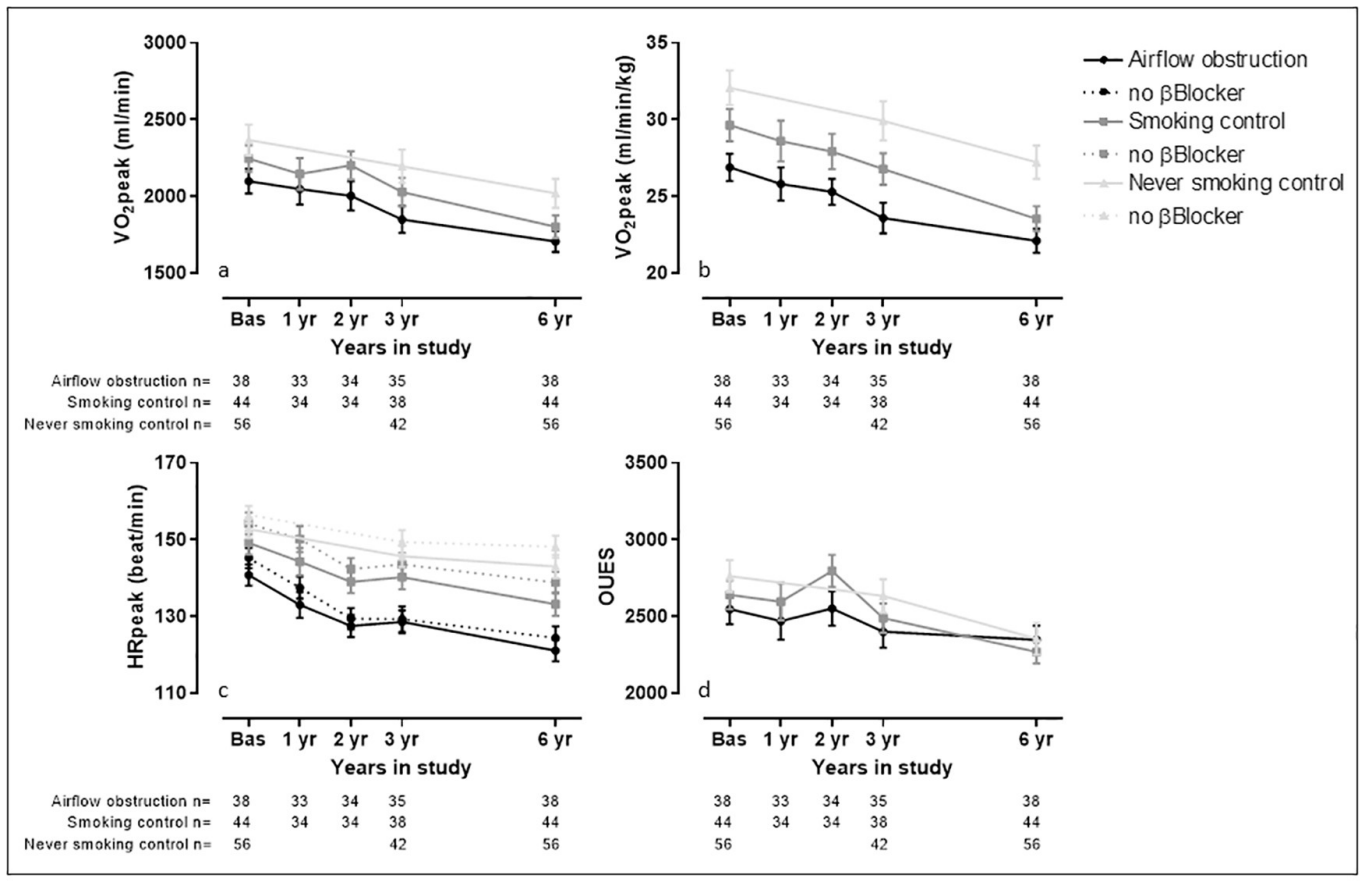
Visual representation of the comprehensive measures of cardiovascular fitness measured during the study. Data are presented as average and standard deviation. Panel a) peak oxygen uptake (VO_2_peak), in mililiters per minute; b) VO_2_peak, in milliliters per minute per kilogram of body weight; c) peak heart rate (HRpeak); d) oxygen uptake efficiency slope (OUES). The Airflow obstruction group is presented by dots and a black line, the smoking control group is presented by squares and a dark grey line and the never smoking control group is presented by triangles and a light grey line. The analysis of HRpeak was performed including all subjects (solid line) and excluding those who were under beta blocker therapy in any of the tests (dotted line) (‘no βBlocker’- Airflow obstruction n = 23, Smoking control n = 32, Never smoking control n = 43).

**Fig 3:**
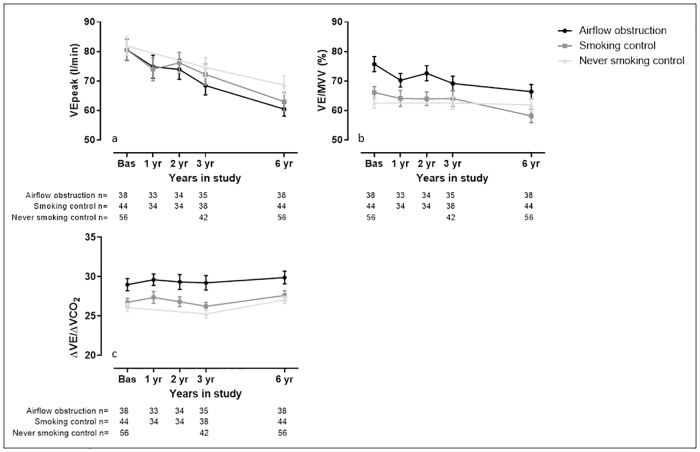
Visual representation of the ventilatory related measures assessed during the study. Data are presented as average and standard deviation. Panel a) peak minute ventilation (VEpeak); b) ventilatory reserve (VE/MVV); c) ventilatory efficiency slope (ΔVE/ΔVCO_2_). The Airflow obstruction group is presented by dots and a black line, the smoking control group is presented by squares and a dark grey line and the never smoking control group is presented by triangles and a light grey line.

**Fig 4:**
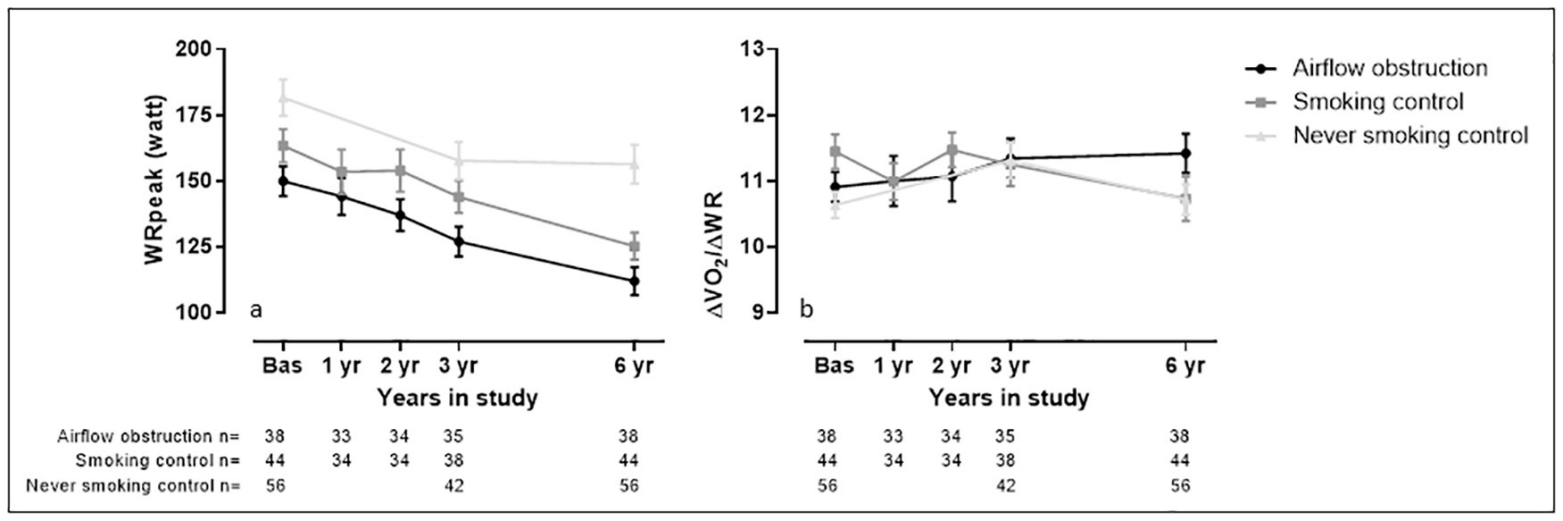
Visual representation of measures related to muscle work assessed during the study. Data are presented as average and standard deviation. Panel a) peak work rate (WRpeak); b) mechanical efficiency (ΔVO_2_/ΔWR). The Airflow obstruction group is presented by dots and a black line, the smoking control group is presented by squares and a dark grey line and the never smoking control group is presented by triangles and a light grey line.

**Fig 5:**
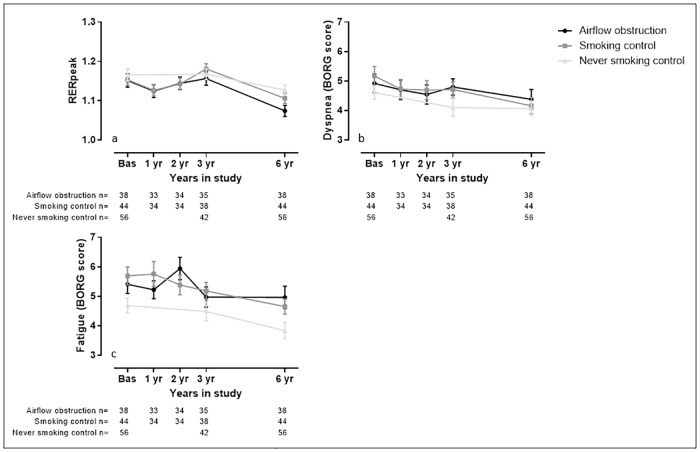
Visual representation of Effort indicators measured during the study. Data presented as average and standard deviation. Panel a) peak respiratory exchange ratio (RERpeak); b) perceived symptoms of dyspnea at the end of the test (BORG score); c) perceived symptoms of fatigue at the end of the test (BORG score). The Airflow obstruction group is presented by dots and a black line, the smoking control group is presented by squares and a dark grey line and the never smoking control group is presented by triangles and a light grey line.

The longitudinal decline of the variables of interest is visually presented in Figs 2–5.

### Exploratory analyses

The introduction of covariates such as age, gender, height and weight in the mixed models did not change the overall data (data not shown).

Subjects from the airflow obstruction group used more frequently respiratory maintenance pharmacotherapy compared to the smoking and never smoking control groups (34% versus 2.3% and 8.9%, p<0.0001). The characteristics of subjects from the airflow obstruction group, subdivided in those who were never or ever under respiratory maintenance pharmacotherapy during follow up, are presented in S4 Table. These baseline characteristics suggest that subjects that ever received maintenance respiratory pharmacotherapy presented statistically worse lung function, diffusion capacity, quadriceps force, functional and maximal exercise capacity, peak ventilation and oxygen efficiency slope. Subjects receiving this type of medication, however, tended to have slower deterioration in exercise capacity compared to patients not receiving maintenance respiratory medication (S5 Table). As groups were small, these differences were not statistically significant (with exception of WRpeak and VE/MVV).

The comparison between subjects who were actively smoking and those who weren’t during the study, among subjects with an important smoking history, can be found in S6 Table. Subjects who did not smoke during the follow up presented a statistically slower deterioration in HRpeak (p = 0.03). No other differences were observed.

Our cohorts presented a considerable amount of cardiovascular and metabolic risk factors and mild comorbidities. The most prevalent at baseline was dyslipidemia, followed by hypertension and pre diabetes (70, 61 and 40% of total sample, respectively). None of risk factors and comorbidities had a statistically different distribution among groups. Only elevated risk for cardiac dysfunction and peripheral arterial disease increased in frequency at six years compared to baseline. This was seen in all groups (data not shown). The rate of decline (slopes) in exercise related variables from those subjects presenting cardiac dysfunction or peripheral arterial disease against those free of these comorbidities for the duration of the study was only statistically significant in the group presenting cardiac dysfunction, which had faster worsening of ventilatory efficiency (yearly change in ΔVE/ΔVCO_2_ = 0.27±0.44 versus 0.05±0.48, p = 0.01) (S7 Table). The group presenting peripheral arterial disease had a trend for a faster deterioration in ventilatory efficiency (yearly change in ΔVE/ΔVCO_2_ = 0.31±0.39 versus 0.10±0.48, p = 0.06). Finally, the comparison of subjects with elevated risk for cardiac dysfunction (n = 21 with NT-proBNP of 205±96) and those with normal values (n = 117 with NT-proBNP of 53±32) showed an impaired OUES in the first group (OUES = 2278±442 versus 2727±701, p<0.01). The deterioration in exercise related variables between the groups at risk or not at baseline was not statistically different and can also be found in S7 Table.

## Discussion

To the best of our knowledge, this is the first study reporting on the long term changes in variables obtained from repeated maximal incremental exercise testing in subjects with mild to moderate airflow obstruction. Indeed, this is the largest study of its kind investigating over 526 exercise tests in never smokers, and (ex-) smokers with and without airflow obstruction. The results did not confirm our hypothesis and the general concept that subjects with mild to moderate airflow obstruction are characterized by a more rapid decline in exercise capacity. This was supported by effort dependent and effort independent outcomes.

Ageing causes an expected deterioration in exercise capacity [29, 30]. Fleg et al. [30] elegantly reported a longitudinal deterioration in VO_2_peak of approximately 2% per year in healthy subjects aged 60 years old. In patients with moderate to severe COPD, Oga et al. [16] reported a yearly rate of decline in VO_2_peak of 32 ml/min, in patients with a baseline of 800 ml/min (approximately 4% per year). More recently, Frisk et al. [17] presented a yearly decline of 50 ml/min in a cohort with somewhat more preserved baseline VO_2_peak (1570 ml/min) (approximately 3.2% per year). These estimated rates are slightly faster compared to that of healthy population reported by Fleg et al. [30]. Our cohort with mild to moderate airflow obstruction presented a yearly deterioration in VO_2_peak of 3±2%, which is similar to the reported by Frisk et al. [17] in their cohort of more severe patients. The rate of deterioration in our study, however, was not statistically faster in subjects with mild to moderate airflow obstruction when compared to never smoking controls.

The oxygen uptake efficiency slope has been proposed as a non-effort dependent marker of fitness [7]. In contrast to our hypothesis, the yearly deterioration in OUES was less pronounced in the airflow obstruction group compared to never and smoking controls (p = 0.03 for both). OUES is highly correlated to the VO_2_peak in cross sectional studies [9] and the percentage of decline of both variables in the never smoking control group was very similar (-2.4% for VO_2_peak versus -2.3% for OUES). However, in the smoking control and airflow obstruction groups, the percentage of decline was somewhat higher for VO_2_peak compared to the effort independent OUES (-3.3% versus -2.3% in smoking control and -3.0% versus -1.1% in the airflow obstruction). This is probably reflecting the effect of a decreased motivation over time on the VO_2_peak decline.

At baseline, although VO_2_peak was lower in subjects with mild to moderate airflow obstruction when compared to the controls, OUES was similar among the groups. Similarly, Terziyski et al. [31] did not find an impaired OUES in the COPD group whereas this was the case in patients with chronic heart disease. In line with this, in the current study, subjects with elevated values of NT-proBNP, a marker for cardiac dysfunction, also presented an impaired OUES compared to those with normal values for NT-proBNP. This might indicate that even mild cardiac comorbidity with a possible impact on cardiac output, affects OUES. The findings of Barron et al. [32] further support this as OUES was strongly able to discriminate patients with heart failure with reduced ejection fraction from healthy subjects, while COPD was discriminated from health by a lower ventilatory reserve but not by differences in OUES. Our data further suggest that the baseline levels of NT-proBNP, however, did not seem to influence the change of OUES over time (S7 Table).

The deterioration in HRpeak was faster in the mild to moderate airflow obstruction group than in the never smoking controls (p<0.01) and in all groups the deterioration was faster than what was described in the eight years longitudinal study of Fleg et al. [30]. Importantly, these effort dependent variables should be interpreted with caution. Since the RERpeak in all groups decreased, it is likely that, overall, the motivation to perform an all-out test decreased, despite the standard encouragement provided by the assessors during the test. This makes it all the more important to look into non- (or less) effort dependent outcomes to evaluate disease progression. At baseline, smoking control subjects had slightly higher scores for fatigue compared to never smoking controls. It is unclear what could have caused this difference. Overall, symptoms of fatigue decreased over time, which might be a reflection of the slight reduction in effort during the maximal test. At the six years time point, symptoms were comparable in all groups, but in the group with airflow obstruction and in the smoking control these symptoms were observed at lower exercise intensities.

Peak pulmonary ventilation decreased over time in all groups. Ventilatory efficiency (ΔVE/ΔVCO_2_) did not deteriorate more rapidly in patients with airflow obstruction. The elevated baseline ΔVE/ΔVCO_2_ was, therefore, maintained for the duration of the study. No longitudinal data exist on the progression of gas exchange abnormalities. The rate of decline in ΔVE/ΔVCO_2_ in the never smoking controls in the present study was comparable to that expected from a cross sectional study in healthy subjects [33]. In our smokers with and without airflow obstruction, the decrease in ventilatory efficiency (increase in ΔVE/ΔVCO_2_) was somewhat less pronounced. Our exploratory analyses indicated a faster deterioration of ΔVE/ΔVCO_2_ in those subjects who presented elevated risk of cardiac dysfunction at baseline or at six years compared to those with normal levels of NTproBNP for the duration of the study. Interestingly, in patients with heart failure, the ΔVE/ΔVCO_2_ is evidenced as an important prognostic marker [34].

Finally, the ΔVO_2_/ΔWR did not change significantly in any group, nor were there differences observed between groups in the deterioration. The estimated means ranged between 10.5 and 11.5, on the edges of the expected range from 8.5 to 10 ml/min increase in VO_2_ for each watt increased in imposed exercise load as proposed in the ATS/ERS statement on exercise testing [6]. To the best of our knowledge, no longitudinal data are available on mechanical efficiency during CPET. Our study suggests that mechanical efficiency remains reasonably stable within an age window of six years.

As previously mentioned, the efficiency slopes are relevant exercise related measures and have the advantage of providing insight in the submaximal exercise responses. These slopes are derived from the relation between two individual variables and the interpretation of their changes might be challenging. For instance, when a patient has the same degree of deterioration on both VO_2_peak and WRpeak, he or she has lost peak aerobic capacity and power, but the mechanical efficiency slope (ΔVO_2_/ΔWR) remains stable. Peak exercise responses, therefore, represent different physiological concepts compared to the slopes. The present study provides data on all relevant peak and slope changes over time.

Exercise capacity is the product of the synergic work from the cardiovascular, respiratory and muscular systems [35]. Patients with advanced COPD often present a dysfunction in these three compartments, which ultimately leads to a decreased exercise capacity compared to healthy peers [4]. As our subjects with mild to moderate airflow obstruction did not present faster rate of deterioration in the exercise related variables compared to the control group, two possible hypotheses remain: 1) Patients with advanced COPD never had an exercise capacity comparable to non COPD peers, or 2) the lower values in severe COPD are the consequence of consecutive acute events such as acute exacerbations that may occur throughout the disease process.

Overall, against our hypothesis, the longitudinal deterioration of effort dependent and effort independent exercise related variables was not faster in our cohort of mild to moderate airflow obstruction. Patients did present with some impairment in exercise capacity at baseline compared to never smoking controls, which was still present at six years. They had lower VO_2_peak, together with lower WRpeak, HRpeak and higher VE/MVV and ΔVE/ΔVCO_2_ compared to never smoking control subjects. These results suggest that the subjects with mild to moderate airway obstruction from our study presented ventilatory constraints, rather than cardiovascular limitations that limited the maximal exercise at baseline.

Our study has some limitations that needed to be considered. First, it is based on a convenience sample from the Rainbow trial and it lacks power to detect subtle differences in the deterioration rate of exercise capacity. Longer studies with a targeted population could shed further light on the development of the exercise limitation over a COPD trajectory. The feasibility of such studies, however, is limited due to the required personal, financial and time investments, besides volunteers’ compliance.

Second, the significant deterioration in RERpeak limited to certain extend our interpretations of those variables that are effort dependent. Nevertheless, this decrease was of small magnitude and similar among groups. More importantly, this study is the first to report results on non-effort dependent variables.

Third, the fact that the baseline levels of maximal exercise capacity were not matched can also be seen as a limitation. For example, if one group attained lower WRpeak than the other, the lower HRpeak can be interpreted as a direct consequence of performing less work. One could argue that in the hypothetic case of higher attained WRpeak, the HRpeak could also be higher. Our three groups were matched for age, gender distribution and BMI, but they already presented some diversity inherent of their condition of being never smokers and (ex-) smokers without and with airflow obstruction. Furthermore, the mixed model analysis did account for potential random differences in intercept (estimated baseline) and rate of decline.

Fourth, subjects who were ex-smokers or did quit smoking during the study, as well as those who received respiratory maintenance pharmacotherapy during the study were not excluded. Subjects who were smoking during the study had similar results compared to those who were not smoking, with the exception of HRpeak, which deteriorated faster in continuing smokers. The use of respiratory maintenance pharmacotherapy tended to lead to a less accentuated deterioration, although this was not statistically significant. Our study was not powered to investigate the effect of respiratory maintenance pharmacotherapy. Based on our results, a total sample of 116 subjects would be needed to find significant differences between patients with and without respiratory maintenance therapy.

Last, the subjects included in this study are a subsample of the initial cohort included in the Rainbow trial. Subjects lost to follow up had more smoking exposure, worse lung function and exercise capacity than those included in the current analysis. This may have led to an underestimated rate of decline in our study due to a healthy survival effect.

## Conclusion

Our six years follow up study indicates that in uneventful mild to moderate airflow obstruction, deterioration in exercise related variables is not accelerated compared to control subjects. Over time, patients may lose motivation to perform maximal exercise test, but effort independent variables confirm that deterioration of exercise capacity and ventilatory efficiency comparable in patients with mild and sTable COPD over a period of six years.

## Supporting information

S1 Table Comparison of baseline characteristics of subjects from the Rainbow trial, included and excluded in this study. Data are expressed as mean±std, number (%) or median [interquartile range]. BMI = body mass index, kg/m^2^ = kilogram per square meter, 6MWD = six minutes walking distance, Nm = Newton meter, MVPA = moderate to intense activities (above 3 METS), min = minute, FEV_1_ = forced expiratory volume in one second, TL,CO = diffusion capacity for carbon monoxide, ml/min/kPa = milliliter per minute per kilopascal, FRC = functional residual capacity, VO_2_peak = peak oxygen uptake, ml/min/kg = milliliter per minute per kilogram, HRpeak = peak heart rate, ‘no βBlocker’ refers to the subgroups of subjects who were not under beta blocker medication at any of the visits (n = 98 in the sample included in the study and n = 63 in the excluded sample), OUES = oxygen efficiency slope, VEpeak = peak minute ventilation, VE/MVV = ventilatory reserve, ΔVE/ΔVCO_2_ = ventilatory efficiency slope, WRpeak = peak work rate, ΔVO_2_/ΔWR = mechanical efficiency, RERpeak = peak respiratory exchange ratio. Missing values: included in the study– 2 for TL,CO, 3 for FRC, 4 for 6MWD, 5 for quadriceps force and symptoms and 9 for physical activity; excluded from the study– 1 for FEV_1_, TL,CO, FRC and 6MWD, 3 for quadriceps force, 6 for physical activity, 10 for CPET variables and 6 for symptoms (from those who performed CPET).
(DOCX)

S2 Table Comparison of baseline characteristics of subjects who were ever and who were never under beta blocker therapy. Data are expressed as mean±std, number (%) or median [interquartile range]. BMI = body mass index, kg/m^2^ = kilogram per square meter, 6MWD = six minutes walking distance, Nm = Newton meter, MVPA = moderate to intense activities (above 3 METS), min = minute, FEV_1_ = forced expiratory volume in one second, TL,CO = diffusion capacity for carbon monoxide, ml/min/kPa = milliliter per minute per kilopascal, FRC = functional residual capacity, VO_2_peak = peak oxygen uptake, ml/min/kg = milliliter per minute per kilogram, HRpeak = peak heart rate, OUES = oxygen efficiency slope, VEpeak = peak minute ventilation, VE/MVV = ventilatory reserve, ΔVE/ΔVCO_2_ = ventilatory efficiency slope, WRpeak = peak work rate, ΔVO_2_/ΔWR = mechanical efficiency, RERpeak = peak respiratory exchange ratio. From the subjects who were ever under beta blocker medication, 14 were in the airflow obstruction group, 12 in the smoking control and 13 in the never smoking control group. Missing values: never under beta blocker medication– 2 for TL,CO and FRC, 3 for 6MWD, 4 for quadriceps force, 6 for physical activity and 4 for symptoms. Ever under beta blocker medication– 1 for 6MWD and FRC, quadriceps force and symptoms, and 3 for physical activity.
(DOCX)

S3 Table Yearly change in absolute and in percentage of estimated baseline for gas exchange, peak minute ventilation and heart rate and ratio between peak minute ventilation and maximal voluntary ventilation. Data are expressed as mean estimate±SD; VO_2_peak = peak oxygen uptake, ml/min/kg = milliliter per minute per kilogram, HRpeak = peak heart rate, ‘no βBlocker’ refers to the subgroups of subjects who were not under beta blocker medication at any of the visits (Airflow obstruction n = 23, Smoking control n = 32, Never smoking control n = 43), OUES = oxygen efficiency slope, VEpeak = peak minute ventilation, VE/MVV = ventilatory reserve, ΔVE/ΔVCO_2_ = ventilatory efficiency slope, WRpeak = peak work rate, ΔVO_2_/ΔWR = mechanical efficiency, RERpeak = peak respiratory exchange ratio. ^¥^ = statistically significant yearly change; ^#^ = statistically different from airflow obstruction; † = statistically different from smoking control; ^₤^ = post hoc indicates a trend for statistically significant difference (p = 0.05) between never smoking control and airflow obstruction groups.
(DOCX)

S4 Table Comparison of baseline characteristics of subjects, from the airflow obstruction group, who were ever and who were never under respiratory maintenance pharmacotherapy. Data are expressed as mean±std, number (%) or median [interquartile range]. BMI = body mass index, kg/m^2^ = kilogram per square meter, 6MWD = six minutes walking distance, Nm = Newton meter, MVPA = moderate to intense activities (above 3 METS), min = minute, FEV_1_ = forced expiratory volume in one second, TL,CO = diffusion capacity for carbon monoxide, ml/min/kPa = milliliter per minute per kilopascal, FRC = functional residual capacity, VO_2_peak = peak oxygen uptake, ml/min/kg = milliliter per minute per kilogram, HRpeak = peak heart rate, ‘no βBlocker’ refers to the subgroups of subjects who were not under beta blocker medication at any of the visits (n = 15 in the sample never under respiratory maintenance pharmacotherapy and n = 8 in the sample ever under respiratory maintenance pharmacotherapy), OUES = oxygen efficiency slope, VEpeak = peak minute ventilation, VE/MVV = ventilatory reserve, ΔVE/ΔVCO_2_ = ventilatory efficiency slope, WRpeak = peak work rate, ΔVO_2_/ΔWR = mechanical efficiency, RERpeak = peak respiratory exchange ratio. Missing values: Subjects who were never under respiratory maintenance pharmacotherapy– 1 for 6MWD and symptoms.
(DOCX)

S5 Table Comparison of the deterioration in exercise related variables between subjects from the airflow obstruction group who were never or ever under respiratory maintenance pharmacotherapy during the follow up period. Data are expressed as mean estimate±SD; VO_2_peak = peak oxygen uptake, ml/min/kg = milliliter per minute per kilogram, HRpeak = peak heart rate, ‘no βBlocker’ refers to the subgroups of subjects who were not under beta blocker medication at any of the visits (n = 15 in the sample never under respiratory maintenance pharmacotherapy and n = 8 in the sample ever under respiratory maintenance pharmacotherapy), OUES = oxygen efficiency slope, VEpeak = peak minute ventilation, VE/MVV = ventilatory reserve, ΔVE/ΔVCO_2_ = ventilatory efficiency slope, WRpeak = peak work rate, ΔVO_2_/ΔWR = mechanical efficiency, RERpeak = peak respiratory exchange ratio. ^¥^ = statistically significant yearly change.
(DOCX)

S6 Table Comparison of the deterioration in exercise related variables between subjects with an important smoking history (either from airflow obstruction or smoking control groups) who were or were not smoking during the follow up. Data are expressed as mean estimate±SD; VO2peak = peak oxygen uptake, ml/min/kg = milliliter per minute per kilogram, HRpeak = peak heart rate, ‘no βBlocker’ refers to the subgroups of subjects who were not under beta blocker medication at any of the visits (n = 25 in ‘not smoking during the study’ and n = 30 in ‘smoking during the study’), OUES = oxygen efficiency slope, VEpeak = peak minute ventilation, VE/MVV = ventilatory reserve, ΔVE/ΔVCO2 = ventilatory efficiency slope, WRpeak = peak work rate, ΔVO2/ΔWR = mechanical efficiency, RERpeak = peak respiratory exchange ratio. Not smoking during the study in the airflow obstruction group n = 17, in the smoking control group n = 24. ¥ = statistically significant yearly change.
(DOCX)

S7 Table Comparison of the deterioration in exercise related variables between subjects with normal or elevated blood levels of NT-proBNP at baseline and between those with normal levels of NT-proBNP for the duration of the study compared to those with elevated levels at baseline or at the end of the follow-up (shaded background), independently of group allocation. Data are expressed as mean estimate±SD; VO2peak = peak oxygen uptake, ml/min/kg = milliliter per minute per kilogram, HRpeak = peak heart rate, ‘no βBlocker’ refers to the subgroups of subjects who were not under beta blocker medication at any of the visits (n = 87 in ‘normal level NT- proBNP’ and n = 10 in ‘elevated NT-proBNP level’ at baseline / n = 72 in ‘normal level NT- proBNP’ and n = 26 in ‘elevated NT-proBNP level’ at baseline or at 6 years), OUES = oxygen efficiency slope, VEpeak = peak minute ventilation, VE/MVV = ventilatory reserve, ΔVE/ΔVCO_2_ = ventilatory efficiency slope, WRpeak = peak work rate, ΔVO_2_/ΔWR = mechanical efficiency, RERpeak = peak respiratory exchange ratio. Elevated NT-proBNP level at baseline in the airflow obstruction group n = 9, in the smoking control n = 4 and in the never smoking control group n = 8. Elevated NT-proBNP level at baseline or at 6 years in the airflow obstruction group n = 17, in the smoking control n = 13 and in the never smoking control group n = 17. ¥ = statistically significant yearly change.
(DOCX)
