# Supplementary material for: Six years progression of exercise capacity in subjects with mild to moderate airflow obstruction, smoking and never smoking controls
Source: PLoS One. 2018 Dec 26;13(12):e0208841. doi: 10.1371/journal.pone.0208841 (PMC6306213; doi:10.1371/journal.pone.0208841)
Supplement: S1 Table — Data are expressed as mean±std, number (%) or median [interquartile range]. BMI = body mass index, kg/m2 = kilogram per square meter, 6MWD = six minutes walking distance, Nm = Newton meter, MVPA = moderate to intense activities (above 3 METS), min = minute, FEV1 = forced expiratory volume in one second, TL,CO = diffusion capacity for carbon monoxide, ml/min/kPa = milliliter per minute per kilopascal, FRC = functional residual capacity, VO2peak = peak oxygen uptake, ml/min/kg = milliliter per minute per kilogram, HRpeak = peak heart rate, ‘no βBlocker’ refers to the subgroups of subjects who were not under beta blocker medication at any of the visits (n = 98 in the sample included in the study and n = 63 in the excluded sample), OUES = oxygen efficiency slope, VEpeak = peak minute ventilation, VE/MVV = ventilatory reserve, ΔVE/ΔVCO2 = ventilatory efficiency slope, WRpeak = peak work rate, ΔVO2/ΔWR = mechanical efficiency, RERpeak = peak respiratory exchange ratio. Missing values: included in the study– 2 for TL,CO, 3 for FRC, 4 for 6MWD, 5 for quadriceps force and symptoms and 9 for physical activity; excluded from the study– 1 for FEV1, TL,CO, FRC and 6MWD, 3 for quadriceps force, 6 for physical activity, 10 for CPET variables and 6 for symptoms (from those who performed CPET). (DOCX) [file pone.0208841.s001.docx]

S1 table. Comparison of baseline characteristics of subjects from the Rainbow trial, included and excluded in this study.

|  | Included (n=138) | Excluded  (n=63) | T test  p |
| --- | --- | --- | --- |
| Age (years) | 62±6 | 62±9 | 0.57 |
| Gender [n (% men)] | 87 (63) | 39 (62) | 0.88 |
| BMI (kg/m^2^) | 26±4 | 27±4 | 0.07 |
| Body weight (kilogram) | 76±14 | 79±13 | 0.22 |
| Smoking hystory (packyear) | 24±26 | 34±24 | 0.02 |
| Not smoking during study [n (%)] | 97 (70) | 30 (48) | <0.01 |
| 6MWD (meter) | 635±75 | 598±96 | <0.01 |
| 6MWD (% predicted) | 96±10 | 92±13 | 0.02 |
| Quadriceps force (Nm) | 159±44 | 153±41 | 0.34 |
| Quadriceps force (% predicted) | 104±24 | 95±19 | 0.02 |
| Steps per day | 9628±3515 | 8423±4189 | 0.04 |
| Time in MVPA (min) | 109±62 | 93±67 | 0.10 |
| Lung function |  |  |  |
| FEV_1_ (liter) | 3.04±0.76 | 2.71±0.76 | <0.01 |
| FEV_1_ (% predicted) | 105±19 | 95±22 | <0.001 |
| TL,CO (ml/min/kPa) | 7.76±2.00 | 7.30±1.90 | 0.13 |
| TL,CO (% predicted) | 89±17 | 84±17 | 0.09 |
| FRC (liter) | 3.73±0.82 | 3.84±0.91 | 0.41 |
| FRC (% predicted) | 115±21 | 118±25 | 0.34 |
| Cardiovascular Fitness |  |  |  |
| VO_2_peak (ml/min) | 2255±644 | 2102±492 | 0.12 |
| VO_2_peak (ml/min/kg) | 30±8 | 27±6 | <0.01 |
| VO_2_peak (% predicted) | 123±33 | 117±29 | 0.26 |
| HRpeak (beats/min) | 148±19 | 136±23 | <0.001 |
| HRpeak (beats/min) - no βBlocker | 153±15 | 136±23 | <0.0001 |
| OUES (slope) | 2665±690 | 2586±600 | 0.46 |
| Pulmonary ventilation |  |  |  |
| VEpeak (l/min) | 81±23 | 76±21 | 0.16 |
| VE/MVV (%) | 67±15 | 72±18 | 0.09 |
| ∆VE/∆VCO_2_ (slope) | 27.08±4.21 | 27.74±4.36 | 0.34 |
| Muscle work |  |  |  |
|  |  |  |  |
| WRpeak (watt) | 167±46 | 144±35 | <0.01 |
| WRpeak (% predicted) | 112±32 | 95±24 | <0.001 |
| ∆VO_2_/∆WR (slope) | 10.97±1.59 | 11.20±1.53 | 0.37 |
| Effort indicators |  |  |  |
| RERpeak | 1.16±0.11 | 1.13±0.09 | 0.14 |
| Symptoms (BORG score) | 6 [4-7] | 5 [4-7] | 0.95 |

Data are expressed as mean±std, number (%) or median [interquartile range]. BMI= body mass index, kg/m^2^ = kilogram per square meter, 6MWD= six minutes walking distance, Nm= Newton meter, MVPA = moderate to intense activities (above 3 METS), min = minute, FEV_1_= forced expiratory volume in one second, TL,CO = diffusion capacity for carbon monoxide, ml/min/kPa = milliliter per minute per kilopascal, FRC= functional residual capacity, VO_2_peak= peak oxygen uptake, ml/min/kg= milliliter per minute per kilogram, HRpeak= peak heart rate, ‘no βBlocker’ refers to the subgroups of subjects who were not under beta blocker medication at any of the visits (n= 98 in the sample included in the study and n=63 in the excluded sample), OUES= oxygen efficiency slope, VEpeak= peak minute ventilation, VE/MVV= ventilatory reserve, ∆VE/∆VCO_2_ = ventilatory efficiency slope, WRpeak= peak work rate, ∆VO_2_/∆WR = mechanical efficiency, RERpeak= peak respiratory exchange ratio. Missing values: included in the study – 2 for TL,CO, 3 for FRC, 4 for 6MWD, 5 for quadriceps force and symptoms and 9 for physical activity; excluded from the study – 1 for FEV_1_, TL,CO, FRC and 6MWD, 3 for quadriceps force, 6 for physical activity, 10 for CPET variables and 6 for symptoms (from those who performed CPET).
