# Supplementary material for: Six years progression of exercise capacity in subjects with mild to moderate airflow obstruction, smoking and never smoking controls
Source: PLoS One. 2018 Dec 26;13(12):e0208841. doi: 10.1371/journal.pone.0208841 (PMC6306213; doi:10.1371/journal.pone.0208841)
Supplement: S2 Table — Data are expressed as mean±std, number (%) or median [interquartile range]. BMI = body mass index, kg/m2 = kilogram per square meter, 6MWD = six minutes walking distance, Nm = Newton meter, MVPA = moderate to intense activities (above 3 METS), min = minute, FEV1 = forced expiratory volume in one second, TL,CO = diffusion capacity for carbon monoxide, ml/min/kPa = milliliter per minute per kilopascal, FRC = functional residual capacity, VO2peak = peak oxygen uptake, ml/min/kg = milliliter per minute per kilogram, HRpeak = peak heart rate, OUES = oxygen efficiency slope, VEpeak = peak minute ventilation, VE/MVV = ventilatory reserve, ΔVE/ΔVCO2 = ventilatory efficiency slope, WRpeak = peak work rate, ΔVO2/ΔWR = mechanical efficiency, RERpeak = peak respiratory exchange ratio. From the subjects who were ever under beta blocker medication, 14 were in the airflow obstruction group, 12 in the smoking control and 13 in the never smoking control group. Missing values: never under beta blocker medication– 2 for TL,CO and FRC, 3 for 6MWD, 4 for quadriceps force, 6 for physical activity and 4 for symptoms. Ever under beta blocker medication– 1 for 6MWD and FRC, quadriceps force and symptoms, and 3 for physical activity. (DOCX) [file pone.0208841.s002.docx]

S2 table. Comparison of baseline characteristics of subjects who were ever and who were never under beta blocker therapy.

|  | Never under beta blocker medication  (n=98) | Ever under beta  blocker medication  (n=40) | T test  p |
| --- | --- | --- | --- |
| Age (years) | 61±6 | 62±6 | 0.34 |
| Gender [n (% men)] | 61 (62) | 26 (65) | 0.76 |
| BMI (kg/m^2^) | 25±3 | 27±4 | <0.01 |
| Body weight (kilogram) | 74±13 | 80±16 | 0.02 |
| Smoking hystory (packyear) | 24±26 | 27±28 | 0.53 |
| Not smoking during study [n (%)] | 68 (69) | 29 (72) | 0.72 |
| 6MWD (meter) | 639±71 | 622±83 | 0.23 |
| 6MWD (% predicted) | 96±10 | 96±12 | 0.91 |
| Quadriceps force (Nm) | 158±42 | 163±50 | 0.52 |
| Quadriceps force (% predicted) | 104±22 | 104±28 | 0.94 |
| Steps per day | 9930±3442 | 8875±3628 | 0.12 |
| Time in MVPA (minutes) | 119±63 | 86±55 | <0.01 |
| Lung function |  |  |  |
| FEV_1_ (liter) | 3.11±0.77 | 2.87±0.72 | 0.09 |
| FEV_1_ (% predicted) | 107±18 | 101±22 | 0.07 |
| TL,CO (ml/min/kPa) | 7.80±2.00 | 7.66±2.04 | 0.71 |
| TL,CO (% predicted) | 89±17 | 87±17 | 0.54 |
| FRC (liter) | 3.75±0.80 | 3.68±0.88 | 0.63 |
| FRC (% predicted) | 116±23 | 112±17 | 0.30 |
| Cardiovascular fitness |  |  |  |
| VO_2_peak (ml/min) | 2296±604 | 2153±730 | 0.24 |
| VO_2_peak (ml/min/kg) | 31±7 | 27±7 | <0.01 |
| VO_2_peak (% predicted) | 125±32 | 117±35 | 0.20 |
| HRpeak (beats/min) | 153±15 | 137±22 | <0.0001 |
| OUES (slope) | 2725±672 | 2518±721 | 0.11 |
| Pulmonary ventilation |  |  |  |
| VEpeak (l/min) | 82±22 | 78±25 | 0.30 |
| VE/MVV (%) | 68±15 | 67±15 | 0.73 |
| ∆VE/∆VCO_2_ (slope) | 26.92±4.42 | 27.47±3.68 | 0.49 |
| Muscle work |  |  |  |
| WRpeak (watt) | 172±46 | 156±46 | 0.06 |
| WR (% predicted) | 115±29 | 104±38 | <0.05 |
| ∆VO_2_/∆WR (slope) | 11.06±1.62 | 10.75±1.51 | 0.30 |
| Effort indicators |  |  |  |
| RERpeak | 1.16±0.10 | 1.16±0.12 | 0.95 |
| Symptoms (BORG score) | 6[5-7] | 5[4-7] | 0.25 |

Data are expressed as mean±std, number (%) or median [interquartile range]. BMI= body mass index, kg/m^2^ = kilogram per square meter, 6MWD= six minutes walking distance, Nm= Newton meter, MVPA = moderate to intense activities (above 3 METS), min = minute, FEV_1_= forced expiratory volume in one second, TL,CO = diffusion capacity for carbon monoxide, ml/min/kPa = milliliter per minute per kilopascal, FRC= functional residual capacity, VO_2_peak= peak oxygen uptake, ml/min/kg= milliliter per minute per kilogram, HRpeak= peak heart rate, OUES= oxygen efficiency slope, VEpeak= peak minute ventilation, VE/MVV= ventilatory reserve, ∆VE/∆VCO_2_ = ventilatory efficiency slope, WRpeak= peak work rate, ∆VO_2_/∆WR = mechanical efficiency, RERpeak= peak respiratory exchange ratio. From the subjects who were ever under beta blocker medication, 14 were in the airflow obstruction group, 12 in the smoking control and 13 in the never smoking control group. Missing values: never under beta blocker medication – 2 for TL,CO and FRC, 3 for 6MWD, 4 for quadriceps force, 6 for physical activity and 4 for symptoms. Ever under beta blocker medication – 1 for 6MWD and FRC, quadriceps force and symptoms, and 3 for physical activity.
