# Supplementary material for: Six years progression of exercise capacity in subjects with mild to moderate airflow obstruction, smoking and never smoking controls
Source: PLoS One. 2018 Dec 26;13(12):e0208841. doi: 10.1371/journal.pone.0208841 (PMC6306213; doi:10.1371/journal.pone.0208841)
Supplement: S3 Table — Data are expressed as mean estimate±SD; VO2peak = peak oxygen uptake, ml/min/kg = milliliter per minute per kilogram, HRpeak = peak heart rate, ‘no βBlocker’ refers to the subgroups of subjects who were not under beta blocker medication at any of the visits (Airflow obstruction n = 23, Smoking control n = 32, Never smoking control n = 43), OUES = oxygen efficiency slope, VEpeak = peak minute ventilation, VE/MVV = ventilatory reserve, ΔVE/ΔVCO2 = ventilatory efficiency slope, WRpeak = peak work rate, ΔVO2/ΔWR = mechanical efficiency, RERpeak = peak respiratory exchange ratio. ¥ = statistically significant yearly change; # = statistically different from airflow obstruction; † = statistically different from smoking control; ₤ = post hoc indicates a trend for statistically significant difference (p = 0.05) between never smoking control and airflow obstruction groups. (DOCX) [file pone.0208841.s003.docx]

S3 table. Yearly change in absolute and in percentage of estimated baseline for gas exchange, peak minute ventilation and heart rate and ratio between peak minute ventilation and maximal voluntary ventilation.

|  | Airflow obstruction | | Smoking control | | Never smoking control | | ANOVA p | |
| --- | --- | --- | --- | --- | --- | --- | --- | --- |
|  | change/year | %change/year | change/year | % change/year | change/year | % change/year | change/year | % change/year |
| Cardiovascular fitness |  |  |  |  |  |  |  |  |
| VO_2_peak (ml/min) | -67±55^¥^ | -3.0±2.2^¥^ | -76±59^¥^ | -3.3±2.3^¥^ | -58±63^¥^ | -2.4±2.6^¥^ | 0.33 | 0.18 |
| VO_2_peak (ml/min/kg) | -0.79±0.69^¥^ | -2.8±2.3^¥^ | -1.04±0.83^¥^ | -3.3±2.4^¥^ | -0.81±0.95^¥^ | -2.4±2.7^¥^ | 0.32 | 0.19 |
| HRpeak (beats/min) | -2.95±2.79^¥^ | -2.0±2.0^¥^ | -2.53±2.25^¥^ | -1.7±1.5^¥^ | -1.62±2.73^¥#^ | -1.0±1.8^¥#^ | 0.04 | 0.02 |
| HRpeak (beats/min) - no βBlocker | -3.18±2.54^¥^ | -2.2±1.8^¥^ | -2.42±2.04^¥^ | -1.5±1.3^¥^ | -1.39±2.24^¥#^ | -0.9±1.4^¥#^ | <0.01 | <0.01 |
| OUES (slope) | -32±62^¥^ | -1.1±2.7^¥^ | -67±63^¥^ | -2.3±2.0^¥^ | -69±88^¥#^ | -2.3±3.0^¥^ | 0.04 | 0.05 |
| Pulmonary ventilation |  |  |  |  |  |  |  |  |
| VEpeak (l/min) | -3.25±2.91^¥^ | -3.7±2.8^¥^ | -2.85±2.37^¥^ | -3.5±2.7^¥^ | -2.22±2.28^¥^ | -2.7±2.6^¥^ | 0.13 | 0.15 |
| VE/MVV (%) | -1.4±2.9^¥^ | -1.5±4.0^¥^ | -1.3±2.4^¥^ | -1.8±3.6^¥^ | -0.11±2.6^₤^ | 0.1±4.4† | 0.03 | 0.03 |
| ∆VE/∆VCO_2_ (slope) | 0.09±0.41 | 0.3±1.3 | 0.11±0.53 | 0.5±1.9 | 0.16±0.48^¥^ | 0.8±1.9¥ | 0.75 | 0.51 |
| Muscle work |  |  |  |  |  |  |  |  |
| WRpeak (watt) | -6.3±4.70^¥^ | -4.0±2.8^¥^ | -6.4±3.94^¥^ | -3.7±2.13^¥^ | -4.2±3.74^¥#^† | -2.5±2.11^¥#^† | 0.01 | <0.01 |
| ∆VO_2_/∆WR (slope) | 0.10±0.35 | 1.2±3.6^¥^ | -0.10±0.46 | -0.6±3.8 | 0.01±0.33 | 0.3±3.0 | 0.07 | 0.06 |
| Effort indicators |  |  |  |  |  |  |  |  |
| RERpeak | -0.011±0.02^¥^ | -0.9±1.4^¥^ | -0.006±0.02^¥^ | -0.5±1.4^¥^ | -0.006±0.02^¥^ | -0.4±1.7 | 0.41 | 0.35 |
| Dyspnea (BORG score) | -0.06±0.38 | - | -0.14±0.39^¥^ | - | -0.09±0.39 | - | 0.60 | - |
| Fatigue (BORG score) | -0.14±0.41^¥^ | - | -0.16±0.35^¥^ | - | -0.11±0.36^¥^ | - | 0.77 | - |

Data are expressed as mean estimate±SD; VO_2_peak= peak oxygen uptake, ml/min/kg= milliliter per minute per kilogram, HRpeak= peak heart rate, ‘no βBlocker’ refers to the subgroups of subjects who were not under beta blocker medication at any of the visits (Airflow obstruction n=23, Smoking control n= 32, Never smoking control n = 43), OUES= oxygen efficiency slope, VEpeak= peak minute ventilation, VE/MVV= ventilatory reserve, ∆VE/∆VCO_2_ = ventilatory efficiency slope, WRpeak= peak work rate, ∆VO_2_/∆WR = mechanical efficiency, RERpeak= peak respiratory exchange ratio. ^¥^= statistically significant yearly change; ^#^= statistically different from airflow obstruction; †= statistically different from smoking control; ^₤^= post hoc indicates a trend for statistically significant difference (p= 0.05) between never smoking control and airflow obstruction groups.
