# Supplementary material for: Six years progression of exercise capacity in subjects with mild to moderate airflow obstruction, smoking and never smoking controls
Source: PLoS One. 2018 Dec 26;13(12):e0208841. doi: 10.1371/journal.pone.0208841 (PMC6306213; doi:10.1371/journal.pone.0208841)
Supplement: S4 Table — Data are expressed as mean±std, number (%) or median [interquartile range]. BMI = body mass index, kg/m2 = kilogram per square meter, 6MWD = six minutes walking distance, Nm = Newton meter, MVPA = moderate to intense activities (above 3 METS), min = minute, FEV1 = forced expiratory volume in one second, TL,CO = diffusion capacity for carbon monoxide, ml/min/kPa = milliliter per minute per kilopascal, FRC = functional residual capacity, VO2peak = peak oxygen uptake, ml/min/kg = milliliter per minute per kilogram, HRpeak = peak heart rate, ‘no βBlocker’ refers to the subgroups of subjects who were not under beta blocker medication at any of the visits (n = 15 in the sample never under respiratory maintenance pharmacotherapy and n = 8 in the sample ever under respiratory maintenance pharmacotherapy), OUES = oxygen efficiency slope, VEpeak = peak minute ventilation, VE/MVV = ventilatory reserve, ΔVE/ΔVCO2 = ventilatory efficiency slope, WRpeak = peak work rate, ΔVO2/ΔWR = mechanical efficiency, RERpeak = peak respiratory exchange ratio. Missing values: Subjects who were never under respiratory maintenance pharmacotherapy– 1 for 6MWD and symptoms. (DOCX) [file pone.0208841.s004.docx]

S4 table. Comparison of baseline characteristics of subjects, from the airflow obstruction group, who were ever and who were never under respiratory maintenance pharmacotherapy.

|  | Never under respiratory maintenance pharmacotherapy  (n=25) | Ever under respiratory maintenance pharmacotherapy  (n=13) | T test  p |
| --- | --- | --- | --- |
| Age (years) | 63±5 | 63±7 | 0.45 |
| Gender [n (% men)] | 22 (88) | 6 (46) | <0.01 |
| BMI (kg/m^2^) | 27±4 | 25±5 | 0.40 |
| Body weight (kilogram) | 83±12 | 71±14 | 0.01 |
| Smoking hystory (packyear) | 41±18 | 52±26 | 0.12 |
| Not smoking during study [n (%)] | 13 (52) | 4 (31) | 0.21 |
| 6MWD (meter) | 623±67 | 575±81 | 0.06 |
| 6MWD (% predicted) | 93±10 | 90±10 | 0.50 |
| Quadriceps force (Nm) | 175±42 | 139±30 | 0.01 |
| Quadriceps force (% predicted) | 98±19 | 105±32 | 0.40 |
| Steps per day | 8867±3800 | 8375±4430 | 0.72 |
| Time in MVPA (minutes) | 101±66 | 93±82 | 0.74 |
| Lung function |  |  |  |
| FEV_1_ (liter) | 2.89±0.55 | 2.16±0.46 | <0.001 |
| FEV_1_ (% predicted) | 93±14 | 83±14 | 0.04 |
| TL,CO (ml/min/kPa) | 7.92±1.67 | 5.61±0.91 | <0.0001 |
| TL,CO (% predicted) | 87±17 | 70±14 | <0.01 |
| FRC (liter) | 4.39±0.87 | 3.86±0.94 | 0.09 |
| FRC (%predicted) | 125±19 | 127±32 | 0.76 |
| Cardiovascular fitness |  |  |  |
| VO_2_peak (ml/min) | 2274±445 | 1763±433 | <0.01 |
| VO_2_peak (ml/min/kg) | 28±4 | 25±7 | 0.26 |
| VO_2_peak (% predicted) | 111±29 | 114±31 | 0.71 |
| HRpeak (beats/min) | 143±16 | 136±18 | 0.19 |
| HRpeak (beats/min) - no βBlocker | 147±11 | 143±16 | 0.48 |
| OUES (slope) | 2733±631 | 2190±391 | <0.01 |
| Pulmonary ventilation |  |  |  |
| VEpeak (l/min) | 86±21 | 69±20 | 0.02 |
| VE/MVV (%) | 76±15 | 76±17 | 0.97 |
| ∆VE/∆VCO_2_ (slope) | 27.96±3.72 | 30.93±6.00 | 0.07 |
| Muscle work |  |  |  |
| WRpeak (watt) | 164±29 | 122±29 | <0.001 |
| WRpeak (% predicted) | 95±18 | 95±23 | 0.99 |
| ∆VO_2_/∆WR (slope) | 11.05±1.21 | 10.64±1.73 | 0.41 |
| Effort indicators |  |  |  |
| RERpeak | 1.16±0.09 | 1.13±0.12 | 0.46 |
| Symptoms (BORG score) | 7[5-7] | 5[4-7] | 0.62 |

Data are expressed as mean±std, number (%) or median [interquartile range]. BMI= body mass index, kg/m^2^ = kilogram per square meter, 6MWD= six minutes walking distance, Nm= Newton meter, MVPA = moderate to intense activities (above 3 METS), min = minute, FEV_1_= forced expiratory volume in one second, TL,CO = diffusion capacity for carbon monoxide, ml/min/kPa = milliliter per minute per kilopascal, FRC= functional residual capacity, VO_2_peak= peak oxygen uptake, ml/min/kg= milliliter per minute per kilogram, HRpeak= peak heart rate, ‘no βBlocker’ refers to the subgroups of subjects who were not under beta blocker medication at any of the visits (n= 15 in the sample never under respiratory maintenance pharmacotherapy and n = 8 in the sample ever under respiratory maintenance pharmacotherapy), OUES= oxygen efficiency slope, VEpeak= peak minute ventilation, VE/MVV= ventilatory reserve, ∆VE/∆VCO_2_ = ventilatory efficiency slope, WRpeak= peak work rate, ∆VO_2_/∆WR = mechanical efficiency, RERpeak= peak respiratory exchange ratio. Missing values: Subjects who were never under respiratory maintenance pharmacotherapy – 1 for 6MWD and symptoms.
