# Supplementary material for: Six years progression of exercise capacity in subjects with mild to moderate airflow obstruction, smoking and never smoking controls
Source: PLoS One. 2018 Dec 26;13(12):e0208841. doi: 10.1371/journal.pone.0208841 (PMC6306213; doi:10.1371/journal.pone.0208841)
Supplement: S5 Table — Data are expressed as mean estimate±SD; VO2peak = peak oxygen uptake, ml/min/kg = milliliter per minute per kilogram, HRpeak = peak heart rate, ‘no βBlocker’ refers to the subgroups of subjects who were not under beta blocker medication at any of the visits (n = 15 in the sample never under respiratory maintenance pharmacotherapy and n = 8 in the sample ever under respiratory maintenance pharmacotherapy), OUES = oxygen efficiency slope, VEpeak = peak minute ventilation, VE/MVV = ventilatory reserve, ΔVE/ΔVCO2 = ventilatory efficiency slope, WRpeak = peak work rate, ΔVO2/ΔWR = mechanical efficiency, RERpeak = peak respiratory exchange ratio. ¥ = statistically significant yearly change. (DOCX) [file pone.0208841.s005.docx]

S5 table. Comparison of the deterioration in exercise related variables between subjects from the airflow obstruction group who were never or ever under respiratory maintenance pharmacotherapy during the follow up period.

|  | Never under respiratory maintenance pharmacotherapy  (n=25) | | Ever under respiratory maintenance pharmacotherapy  (n=13) | | T test p | |
| --- | --- | --- | --- | --- | --- | --- |
|  | change/year | %change/year | change/year | % change/year | change/year | % change/year |
| Cardiovascular fitness |  |  |  |  |  |  |
| VO_2_peak (ml/min) | -76±55^¥^ | -3.2±2.1^¥^ | -48±51^¥^ | -2.6±2.5^¥^ | 0.14 | 0.50 |
| VO_2_peak (ml/min/kg) | -0.86±0.65^¥^ | -3.0±2.1^¥^ | -0.66±0.76^¥^ | -2.5±2.7^¥^ | 0.39 | 0.55 |
| HRpeak (beats/min) | -3.15±2.69^¥^ | -2.2±1.8^¥^ | -2.56±3.06^¥^ | -1.8±2.2^¥^ | 0.55 | 0.64 |
| HRpeak (beats/min) - no βBlocker | -3.43±1.62^¥^ | -2.4±1.0^¥^ | -2.72±3.82 | -1.8±2.8 | 0.53 | 0.49 |
| OUES (slope) | -40±60^¥^ | -1.3±2.2^¥^ | -17±66 | -0.6±3.5 | 0.27 | 0.41 |
| Pulmonary ventilation |  |  |  |  |  |  |
| VEpeak (l/min) | -8.82±2.78^¥^ | -4.1±2.6^¥^ | -2.17±2.96^¥^ | -2.8±3.2^¥^ | 0.10 | 0.16 |
| VE/MVV (%) | -2.3±2.4^¥^ | -2.8±3.3^¥^ | 0.3±3.1 | 0.9±4.1 | <0.01 | <0.01 |
| ∆VE/∆VCO_2_ (slope) | 0.14±41 | 0.5±1.3 | -0.003±0.42 | 0.07±1.3 | 0.31 | 0.37 |
| Muscle work |  |  |  |  |  |  |
| WRpeak (watt) | -7.59±4.41^¥^ | -4.5±2.4^¥^ | -3.86±4.39^¥^ | -3.0±3.4^¥^ | 0.02 | 0.13 |
| ∆VO_2_/∆WR (slope) | 0.08±0.28 | 0.9±2.5 | 0.13±0.48 | 1.9±5.2 | 0.67 | 0.41 |
| Effort indicators |  |  |  |  |  |  |
| RERpeak | -0.013±0.014^¥^ | -1.1±1.2^¥^ | -0.006±0.020 | -0.5±1.7 | 0.21 | 0.19 |
| Dyspnea (BORG score) | -0.11±0.36 | - | 0.05±0.41 | - | 0.22 | - |
| Fatigue (BORG score) | -0.11±0.44 | - | -0.19±0.36 | - | 0.57 | - |

Data are expressed as mean estimate±SD; VO_2_peak= peak oxygen uptake, ml/min/kg= milliliter per minute per kilogram, HRpeak= peak heart rate, ‘no βBlocker’ refers to the subgroups of subjects who were not under beta blocker medication at any of the visits (n= 15 in the sample never under respiratory maintenance pharmacotherapy and n = 8 in the sample ever under respiratory maintenance pharmacotherapy), OUES= oxygen efficiency slope, VEpeak= peak minute ventilation, VE/MVV= ventilatory reserve, ∆VE/∆VCO_2_ = ventilatory efficiency slope, WRpeak= peak work rate, ∆VO_2_/∆WR = mechanical efficiency, RERpeak= peak respiratory exchange ratio. ^¥^= statistically significant yearly change.
