# Supplementary material for: Six years progression of exercise capacity in subjects with mild to moderate airflow obstruction, smoking and never smoking controls
Source: PLoS One. 2018 Dec 26;13(12):e0208841. doi: 10.1371/journal.pone.0208841 (PMC6306213; doi:10.1371/journal.pone.0208841)
Supplement: S6 Table — Data are expressed as mean estimate±SD; VO2peak = peak oxygen uptake, ml/min/kg = milliliter per minute per kilogram, HRpeak = peak heart rate, ‘no βBlocker’ refers to the subgroups of subjects who were not under beta blocker medication at any of the visits (n = 25 in ‘not smoking during the study’ and n = 30 in ‘smoking during the study’), OUES = oxygen efficiency slope, VEpeak = peak minute ventilation, VE/MVV = ventilatory reserve, ΔVE/ΔVCO2 = ventilatory efficiency slope, WRpeak = peak work rate, ΔVO2/ΔWR = mechanical efficiency, RERpeak = peak respiratory exchange ratio. Not smoking during the study in the airflow obstruction group n = 17, in the smoking control group n = 24. ¥ = statistically significant yearly change. (DOCX) [file pone.0208841.s006.docx]

S6 table. Comparison of the deterioration in exercise related variables between subjects who were or were not smoking during the follow up, from those with an important smoking history.

|  | Not smoking during the study  (n=41) | | Smoking during the study  (n=41) | | T test p | |
| --- | --- | --- | --- | --- | --- | --- |
|  | change/year | %change/year | change/year | % change/year | change/year | % change/year |
| Cardiovascular fitness |  |  |  |  |  |  |
| VO_2_peak (ml/min) | -72±68^¥^ | -2.8±2.6^¥^ | -72±44^¥^ | -3.5±1.9^¥^ | 0.99 | 0.19 |
| VO_2_peak (ml/min/kg) | -0.92±0.86^¥^ | -2.9±2.5^¥^ | -0.93±0.68^¥^ | -3.3±2.2^¥^ | 0.94 | 0.42 |
| HRpeak (beats/min) | -2.23±2.49^¥^ | -1.5±1.7^¥^ | -3.23±2.45^¥^ | -2.2±1.7^¥^ | 0.07 | 0.04 |
| HRpeak (beats/min) - no βBlocker | -2.01±2.33^¥^ | -1.2±1.6^¥^ | -3.36±2.07^¥^ | -2.3±1.4^¥^ | 0.03 | 0.01 |
| OUES (slope) | -54±76^¥^ | -1.6±2.9^¥^ | -48±50^¥^ | -1.8±1.9^¥^ | 0.65 | 0.72 |
| Pulmonary ventilation |  |  |  |  |  |  |
| VEpeak (l/min) | -2.76±2.70^¥^ | -3.1±2.8^¥^ | -3.31±2.55^¥^ | -4.1±2.6^¥^ | 0.35 | 0.11 |
| VE/MVV (%) | -1.3±2.7^¥^ | -1.7±4.0^¥^ | -1.3±2.6^¥^ | -1.7±3.5^¥^ | 0.97 | 0.99 |
| ∆VE/∆VCO_2_ (slope) | 0.10±0.49 | 0.4±1.8 | 0.11±0.47 | 0.5±1.6 | 0.89 | 0.95 |
| Muscle work |  |  |  |  |  |  |
| WRpeak (watt) | -6.42±4.79^¥^ | -3.6±2.6^¥^ | -6.31±3.76^¥^ | -4.1±2.3^¥^ | 0.90 | 0.40 |
| ∆VO_2_/∆WR (slope) | -0.06±0.47 | -0.09±4.2 | 0.04±0.37 | 0.58±3.4 | 0.30 | 0.43 |
| Effort indicators |  |  |  |  |  |  |
| RERpeak | -0.006±0.016^¥^ | -0.4±1.4 | -0.011±0.015^¥^ | -0.9±1.3^¥^ | 0.11 | 0.10 |
| Dyspnea (BORG score) | -0.11±0.45 | - | -0.09±0.31 | - | 0.81 | - |
| Fatigue (BORG score) | -0.16±0.37^¥^ | - | -0.14±0.38^¥^ | - | 0.84 | - |

Data are expressed as mean estimate±SD; VO_2_peak= peak oxygen uptake, ml/min/kg= milliliter per minute per kilogram, HRpeak= peak heart rate, ‘no βBlocker’ refers to the subgroups of subjects who were not under beta blocker medication at any of the visits (n= 25 in ‘not smoking during the study’ and n = 30 in ‘smoking during the study’), OUES= oxygen efficiency slope, VEpeak= peak minute ventilation, VE/MVV= ventilatory reserve, ∆VE/∆VCO_2_ = ventilatory efficiency slope, WRpeak= peak work rate, ∆VO_2_/∆WR = mechanical efficiency, RERpeak= peak respiratory exchange ratio. Not smoking during the study in the airflow obstruction group n = 17, in the smoking control group n = 24. ^¥^= statistically significant yearly change.
