# Supplementary material for: Six years progression of exercise capacity in subjects with mild to moderate airflow obstruction, smoking and never smoking controls
Source: PLoS One. 2018 Dec 26;13(12):e0208841. doi: 10.1371/journal.pone.0208841 (PMC6306213; doi:10.1371/journal.pone.0208841)
Supplement: S7 Table — Data are expressed as mean estimate±SD; VO2peak = peak oxygen uptake, ml/min/kg = milliliter per minute per kilogram, HRpeak = peak heart rate, ‘no βBlocker’ refers to the subgroups of subjects who were not under beta blocker medication at any of the visits (n = 87 in ‘normal level NT- proBNP’ and n = 10 in ‘elevated NT-proBNP level’ at baseline / n = 72 in ‘normal level NT- proBNP’ and n = 26 in ‘elevated NT-proBNP level’ at baseline or at 6 years), OUES = oxygen efficiency slope, VEpeak = peak minute ventilation, VE/MVV = ventilatory reserve, ΔVE/ΔVCO2 = ventilatory efficiency slope, WRpeak = peak work rate, ΔVO2/ΔWR = mechanical efficiency, RERpeak = peak respiratory exchange ratio. Elevated NT-proBNP level at baseline in the airflow obstruction group n = 9, in the smoking control n = 4 and in the never smoking control group n = 8. Elevated NT-proBNP level at baseline or at 6 years in the airflow obstruction group n = 17, in the smoking control n = 13 and in the never smoking control group n = 17. ¥ = statistically significant yearly change. (DOCX) [file pone.0208841.s007.docx]

S7 table. Comparison of the deterioration in exercise related variables between subjects with normal or elevated blood levels of NT-proBNP at baseline and between those with normal levels of NT-proBNP for the duration of the study compared to those with elevated levels at baseline or at the end of the follow-up (shaded background), independently of group allocation.

|  | Normal NT-proBNP level at baseline (n=115) | Elevated NT-proBNP level at baseline (n=21) | T test p | Normal NT-proBNP level at baseline and at 6 years (n=91) | Elevated NT-proBNP level at baseline or at 6 years (n=47) | T test p |
| --- | --- | --- | --- | --- | --- | --- |
| Cardiovascular fitness |  |  |  |  |  |  |
| VO_2_peak (ml/min) | -65±57^¥^ | -73±7.4^¥^ | 0.59 | -63±57^¥^ | -73±65^¥^ | 0.35 |
| VO_2_peak (ml/min/kg) | -0.88±0.80^¥^ | -0.86±1.12^¥^ | 0.90 | -0.86±0.81^¥^ | -0.92±0.93^¥^ | 0.72 |
| HRpeak (beats/min) | -2.31±2.63^¥^ | -2.31±2.79^¥^ | 0.99 | -2.15±2.52^¥^ | -2.53±2.89^¥^ | 0.43 |
| HRpeak (beats/min) - no βBlocker | -2.08±2.37^¥^ | -3.10±1.84^¥^ | 0.19 | -2.03±2.37^¥^ | -2.48±2.29^¥^ | 0.40 |
| OUES (slope) | -58±74^¥^ | -54±82^¥^ | 0.80 | -59±75^¥^ | -58±76^¥^ | 0.94 |
| Pulmonary ventilation |  |  |  |  |  |  |
| VEpeak (l/min) | -2.66±2.45^¥^ | -3.09±2.90^¥^ | 0.47 | -2.71±2.51^¥^ | -2.69±2.56^¥^ | 0.96 |
| VE/MVV (%) | -0.90±2.5^¥^ | -0.70±3.5 | 0.75 | -1.0±2.5^¥^ | -0.5±2.9 | 0.24 |
| ∆VE/∆VCO_2_ (slope) | 0.11±0.48^¥^ | 0.24±0.45^¥^ | 0.24 | 0.05±0.48 | 0.27±0.44^¥^ | 0.01 |
| Muscle work |  |  |  |  |  |  |
| WRpeak (watt) | -5.35±4.16^¥^ | -6.41±4.36^¥^ | 0.29 | -5.23±4.12^¥^ | -5.98±4.33^¥^ | 0.32 |
| ∆VO_2_/∆WR (slope) | 0.02±0.36 | -0.09±0.54 | 0.24 | 0.03±0.36 | -0.07±0.43 | 0.14 |
| Effort indicator |  |  |  |  |  |  |
| RERpeak | -0.008±0.017^¥^ | -0.010±0.023^¥^ | 0.51 | -0.008±0.018^¥^ | -0.007±0.019^¥^ | 0.71 |

Data are expressed as mean estimate±SD; VO_2_peak= peak oxygen uptake, ml/min/kg= milliliter per minute per kilogram, HRpeak= peak heart rate, ‘no βBlocker’ refers to the subgroups of subjects who were not under beta blocker medication at any of the visits (n= 87 in ‘normal level NT- proBNP’ and n = 10 in ‘elevated NT-proBNP level’ at baseline / n = 72 in ‘normal level NT- proBNP’ and n = 26 in ‘elevated NT-proBNP level’ at baseline or at 6 years), OUES= oxygen efficiency slope, VEpeak= peak minute ventilation, VE/MVV= ventilatory reserve, ∆VE/∆VCO_2_ = ventilatory efficiency slope, WRpeak= peak work rate, ∆VO_2_/∆WR = mechanical efficiency, RERpeak= peak respiratory exchange ratio. Elevated NT-proBNP level at baseline in the airflow obstruction group n = 9, in the smoking control n = 4 and in the never smoking control group n=8. Elevated NT-proBNP level at baseline or at 6 years in the airflow obstruction group n = 17, in the smoking control n = 13 and in the never smoking control group n= 17. ^¥^= statistically significant yearly change.
